# ﻿Size matters: a new genus of tarantula with the longest male palps, and an integrative revision of *Monocentropus* Pocock, 1897 (Araneae, Theraphosidae, Eumenophorinae)

**DOI:** 10.3897/zookeys.1247.162886

**Published:** 2025-07-22

**Authors:** Alireza Zamani, Volker von Wirth, Přemysl Fabiánek, Jonas Höfling, Pavel Just, Jan Korba, Alice Petzold, Mark Stockmann, Hassan Sh Abdirahman Elmi, Miguel Vences, Vera Opatova

**Affiliations:** 1 Department of Biology, University of Turku, 20500 Turku, Finland University of Turku Turku Finland; 2 Theraphosid Research Team, Hofmarkstraße 6, 85462 Eitting, Germany Theraphosid Research Team Eitting Germany; 3 Velké Hamry 393, 562 01 Ústí nad Orlicí, Czech Republic Unaffiliated Ústí nad Orlicí Czech Republic; 4 Division of Evolutionary Biology, Zoological Institute, Braunschweig University of Technology, Mendelssohnstr. 4, 38106 Braunschweig, Germany Braunschweig University of Technology Braunschweig Germany; 5 Department of Zoology, Faculty of Science, Charles University, Viničná 7, 128 00 Prague, Czech Republic Charles University Prague Czech Republic; 6 Faculty of Sciences, Institute of Biochemistry and Biology, University of Potsdam, Potsdam, Germany University of Potsdam Potsdam Germany; 7 Museum für Naturkunde, Leibniz Institute for Evolution and Biodiversity Science, Berlin, Germany Museum für Naturkunde, Leibniz Institute for Evolution and Biodiversity Science Berlin Germany; 8 Im Hoek 20, 48477 Hörstel-Riesenbeck, Germany Unaffiliated Hörstel-Riesenbeck Germany; 9 Department of Biology, Faculty of Education, Amoud University, 2526 Borama, Somalia Amoud University Borama Somalia

**Keywords:** Afrotropical realm, Arabian Peninsula, Madagascar, *Satyrex* gen. nov., Somaliland

## Abstract

A taxonomic revision of the eumenophorine tarantula genus *Monocentropus* Pocock, 1897, which currently comprises three species, *M.balfouri* Pocock, 1897 (♂♀; Socotra, Yemen), *M.lambertoni* Fage, 1922 (♂♀; Madagascar), and *M.longimanus* Pocock, 1903 (♂♀; Yemen), is presented. By integrating both morphological data and a molecular phylogeny based on mitochondrial (*cox1*) and nuclear (*28S*, *18S*) markers, the genus is herein redefined to include only the type species, *M.balfouri*. A new genus, *Satyrex* Zamani & von Wirth, **gen. nov.**, is established to comprise *S.longimanus***comb. nov.**, along with four new species from the Arabian Peninsula and the Horn of Africa described herein: *S.arabicus* Zamani & von Wirth, **sp. nov.** (♂; Saudi Arabia), *S.ferox* Zamani, von Wirth & Stockmann, **sp. nov.** (♂♀; Yemen, Oman), *S.somalicus* Zamani & von Wirth, **sp. nov.** (♂; Somaliland), and *S.speciosus* Zamani, von Wirth & Just, **sp. nov.** (♂♀; Somaliland). The new genus is partially characterised by possessing the longest male palps known in tarantulas, possibly functioning in cannibalism avoidance during mating. Both the molecular phylogeny and morphological characters suggest that *M.lambertoni* is probably not congeneric with *M.balfouri*, and also indicate that multiple species may be currently subsumed under the former name. Therefore, *M.lambertoni* is regarded as incerta sedis pending further studies to clarify its taxonomic placement, as it is also considered to represent a species complex. Finally, the distribution of all studied taxa is discussed within a biogeographic framework.

## ﻿Introduction

Theraphosidae Thorell, 1869 is the largest family of mygalomorph spiders, comprising more than 1,140 extant species in 175 genera and 13 or 14 subfamilies (Pérez-Miles 2020; WSC 2025). Commonly known as “tarantulas,” most theraphosids are large, hairy spiders primarily found in tropical and subtropical regions (Pérez-Miles 2020). Three genera of Theraphosidae are known from the Middle East: *Chaetopelma* Ausserer, 1871; *Ischnocolus* Ausserer, 1871; and *Monocentropus* Pocock, 1897. Although *Chaetopelma* and *Ischnocolus* were traditionally considered closely related and both as belonging to the subfamily Ischnocolinae Simon, 1892, a recent integrative analysis by Korba et al. (2022) has proposed that *Chaetopelma* is instead sister to the Afrotropical subfamily Eumenophorinae Pocock, 1897, which includes *Monocentropus*, as well as 12 other genera. *Monocentropus* is a small genus currently comprising three species: *M.balfouri* Pocock, 1897, from Socotra Island, Yemen; *M.lambertoni* Fage, 1922, from Madagascar; and *M.longimanus* Pocock, 1903, from Yemen (WSC 2025). The disjunct distribution of these three species is highly unusual for a small genus of spiders with low mobility. Moreover, the male of *M.longimanus* possesses extraordinarily long palps unknown in any other species of the family, and the morphology of its copulatory bulb significantly differs from that of the other two described congeners. Our examination of a series of specimens representing four undescribed species from the Arabian Peninsula and the Horn of Africa revealed that they also possess elongated male palps and bulbs of similar shape, which indicate that they may be closely related to *M.longimanus*.

In this paper, we employ an integrative approach to revise *Monocentropus*, re-evaluate the generic placement of *M.lambertoni* and *M.longimanus*, and describe four new species. We further hypothesise about the possible function of the elongated male palps in *M.longimanus* and the newly described species. Finally, we discuss the potential cryptic diversity of species closely related to *M.lambertoni* in Madagascar, as well as the distribution of all studied taxa within a biogeographic context.

## ﻿Material and methods

### ﻿Morphological examination and technical data

Photographs of the preserved specimens and their structures were taken using an Olympus Camedia E‐520 camera adapted to an Olympus SZX16 stereomicroscope at the Zoological Museum of the University of Turku, or a Touptek 32 MP USB3.0 CMOS C-Mount camera adapted to a Nikon SMZ18 stereomicroscope at the personal laboratory of the Theraphosid Research Team. Digital images of different focal planes were stacked with Helicon Focus™ v. 8.1.1 and the final images were edited using Adobe Photoshop CS2 9.0. Illustrations of receptacles were made after digesting tissues off in a 10% KOH aqueous or a pepsin enzyme solution (von Wirth and Hildebrandt 2023). Body measurements exclude the chelicerae and spinnerets. Leg segments were measured on the dorsal side. Measurements of palp and legs are listed as: total (femur, patella, tibia, metatarsus [absent in palp], tarsus). The terminology used for the keels of the embolus follows that proposed by Bertani (2000) for Theraphosinae, although it does not reflect homology and is based primarily on position. The abbreviations “p” and “r” for distal ventral metatarsal spines refer to the position of the lateral spines relative to the median spine, whereas for distal tibial spines, they indicate their positions relative to areas of the segment. All measurements are given in millimetres. The map was prepared using SimpleMappr (Shorthouse 2010).

The following abbreviations are used in the text and figures: Keels of the embolus: **A**—apical, **PI**—prolateral inferior, **PS**—prolateral superior. Spination: **m**—median, **p**—prolateral, **r**—retrolateral. Spinnerets: **PLS**—posterior lateral spinneret, **PMS**—posterior median spinneret. Depositories: **NHMUK**—Natural History Museum, London, UK (J. Beccaloni); **MNHN**—Muséum national d'Histoire naturelle, Paris, France (K. Privet); **SMF**—Naturmuseum Senckenberg, Frankfurt am Main, Germany (P. Jäger); **SMNS**—Staatliches Museum für Naturkunde Stuttgart, Germany (I. Wendt).

### ﻿Molecular procedures and phylogenetic analysis

Whole genomic DNA was extracted from eight theraphosid individuals originating from the Arabian Peninsula and the Horn of Africa (see Suppl. material 3), using the DNeasy Blood and Tissue Kit (QIAGEN), following the manufacturer’s protocol. Partial fragments of one mitochondrial gene—cytochrome oxidase I (*cox1*)—and two nuclear genes—28S rDNA (*28S*) and 18S rDNA (*18S*)—were amplified for this study using the following primer combinations: C1-J-1490/C1-N-2198 (Folmer et al. 1994) for *cox1*, 28sO/28sB (Whiting et al. 1997; Hedin and Maddison 2001) for *28S*, and 5R/9R (Whiting et al. 1997) for *18S*. PCR products were purified using the MinElute PCR Purification Kit (QIAGEN) and Sanger-sequenced in both directions by Macrogen Inc. (Amsterdam, the Netherlands). Chromatograms were assembled and edited using Geneious^®^ v. 10.1.3 (Biomatters Ltd., Auckland, New Zealand).

For specimens from Madagascar, sequences of several gene fragments were available from the work of Lüddecke et al. (2018), including material from sites in both rainforest (Marojejy) and a dry karstic islet (Nosy Hara). Due to the limited success of PCR-based approaches for these and other Malagasy samples, we employed a museomics approach based on archival DNA sequencing. In 2025, we sampled the original collection specimens, preserved in approximately 80% ethanol, by extracting small pieces of leg muscle. Each sample was stored in a vial containing pure ethanol. The samples were first weighed and then incubated overnight at 37 °C in a Guanidine Thiocyanate (GuSCN)-based extraction buffer solution. The following day, genomic DNA was extracted in a total volume of 25 µl using the protocol of Rohland et al. (2004), following several sequential steps as described in Straube et al. (2021). DNA yield was quantified from 1 µl of extract using the Qubit dsDNA HS Assay Kit (0.2–100 ng/μl; Life Technologies, Carlsbad, California, USA), according to the manufacturer’s instructions. Up to 13 ng of DNA was used as input for single-stranded library preparation, following the protocol of Gansauge et al. (2017). All laboratory work prior to qPCR was conducted in a dedicated ancient DNA facility at the University of Potsdam, Germany, which complies with all requirements for handling historical samples (see Fulton and Shapiro 2019). Extraction and library blanks were processed alongside all samples to monitor potential contamination. Final library concentrations and fragment length distributions were assessed using a 2200 TapeStation (Agilent Technologies) assay. Libraries were shotgun-sequenced to obtain approximately five million 75-bp single-end reads on an Illumina NextSeq 500/550 platform at the University of Potsdam, as described in Paijmans et al. (2017).

Read quality was visualised twice using FastQC (https://www.bioinformatics.babraham.ac.uk), both before and after adapter trimming and removal of reads shorter than 30 bp using cutadapt v. 1.12 (Martin 2011). We then used local BLAST (BLAST+; Camacho et al. 2009), as implemented in BlasTax, a tool of the iTaxoTools project (Vences et al. 2021), to search against a reference library of all available *18S*, *28S*, and *cox1* sequences of eumenophorine tarantulas from Lüddecke et al. (2018). Reads matching with at least 90% similarity were collected in a FASTA file. These matching reads were then mapped to *Monocentropuslambertoni* reference sequences using CodonCode Aligner v. 3.7.1 (CodonCode Corporation), with gaps between contigs represented by the letter “N”.

Outgroup sequence data (Hedin and Bond 2006; Korba et al. 2022; Leavitt et al. 2015; Lüddecke et al. 2018), representing all Theraphosidae subfamilies (Lüddecke et al. 2018) and related mygalomorph families (Opatova et al. 2020) Barychelidae, Bemmeridae, and Nemesiidae, were obtained from GenBank (Suppl. material 3). Due to the presence of indel mutations, per-locus alignments were performed using the Q-INS-i algorithm in the online version of MAFFT (Katoh et al. 2019). Alignments were visually inspected and concatenated into a single matrix in Geneious.

Maximum likelihood (ML) analysis was conducted in IQ-TREE (Minh et al. 2020). Each partition (*18S*, *28S*, *cox1* 1^st^+2^nd^ positions, and *cox1* 3^rd^ position) was assigned an independent substitution model inferred by ModelFinder (Kalyaanamoorthy et al. 2017). The best ML tree was selected from 1,000 iterations, and node support was assessed using 1,000 ultrafast bootstrap replicates. Bayesian Inference (BI) analysis was conducted in MrBayes v. 3.2.7 (Ronquist et al. 2012) using the same partitioning scheme. Two independent runs of 5 × 10^7^ generations, each with eight MCMC (Markov Chain Monte Carlo) chains starting from random trees and resampling every 1,000 generations, were executed simultaneously. The first 25% of generations were discarded as burn-in. Convergence and chain mixing were assessed by the standard deviation of split frequencies (<0.01), and effective sample sizes (ESS) were summarised using Tracer v. 1.6 (Rambaut et al. 2014). Genetic distances among the lineages from the Arabian Peninsula and the Horn of Africa were calculated based on *cox1* data using uncorrected *p*-distances in MEGA11 (Tamura et al. 2021).

## ﻿Results

### ﻿Taxonomic sampling, sequencing, alignment, and phylogenetic analyses

**Figure 1.**
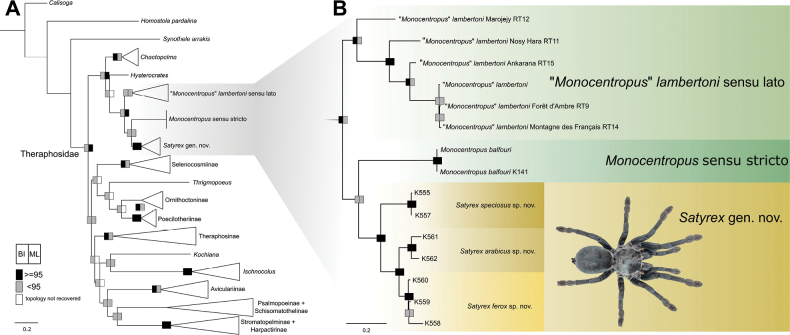
Phylogeny of the family Theraphosidae (**A**), and detailed phylogenetic relationships among “*Monocentropus*” *lambertoni* sensu lato, *Monocentropusbalfouri*, and *Satyrex* gen. nov. (**B**). Both tree topologies were inferred using Bayesian analysis based on three loci (*cox1*, *18S*, *28S*). Node support values from Bayesian inference (posterior probability, PP) and maximum likelihood (ultrafast bootstrap, UFBoot) are indicated by colored boxes: black = supported (PP ≥ 95, UFBoot ≥ 95); grey = not supported (PP < 95, UFBoot < 95); white = topology not recovered. Separate Bayesian and maximum likelihood trees are provided in the Suppl. materials 1, 2). Photo: adult female of *Satyrexferox* sp. nov., by PF.

Specimen information and GenBank accession numbers for genetic data used in this study are provided in Suppl. material 3. The newly sequenced gene fragments targeted in this study yielded the following lengths: *cox1* (550–630 bp), *28S* (550–790 bp) and *18S* (890–1022 bp). The concatenated matrix (2570 bp; *cox1*: 660, *28S*: 838, *18S*: 1070) comprised 72 terminals, representing 15 individuals of *Monocentropus* sensu lato and 57 outgroup taxa (Theraphosidae: 54 terminals, Barychelidae: 1, Bemmeridae: 1, Nemesiidae: 1). The partition scheme and its corresponding substitution models were selected by ModelFinder as follows: *18S*: TNe (K80 in BI); *28S*: GTR + I; 1^st^ + 2^nd^*cox1* position: TIM (HKY in BI); 3^rd^*cox1* position: GTR + I. Both ML and BI analyses yielded topologies (Fig. 1, Suppl. materials 1, 2) with low support values for deeper Theraphosidae nodes. Lineages of *Monocentropus* sensu lato (i.e., “*Monocentropus*” *lambertoni* sensu lato, *Monocentropus* sensu stricto, and *Satyrex* gen. nov., see Taxonomy) formed a clade (supported in ML, Suppl. material 2) in both analyses. The topology of the intra-generic relationships was congruent between the two approaches, but a few nodes received low support values (Suppl. materials 1, 2). Individuals from Madagascar formed a partially supported clade in ML (Suppl. material 2); *M.balfouri* was recovered as a sister lineage to a monophyletic group (supported in both analyses) comprising individuals from the Arabian Peninsula and the Horn of Africa. This group is herein considered as a separate genus, *Satyrex* gen. nov. Mitochondrial genetic divergences among the lineages from the Arabian Peninsula and the Horn of Africa were high, with uncorrected *p*-distances for the *cox1* fragment ranging from 8.6% (between *S.ferox* sp. nov. and *S.arabicus* sp. nov.) to 13.2% (between *S.ferox* sp. nov. and *S.speciosus* sp. nov.) and 14.2% (between *S.arabicus* sp. nov. and *S.speciosus* sp. nov.). No molecular data could be obtained from the holotype of *S.somalicus* sp. nov. due to the poor DNA quality of the specimen. A substantial genetic variation was also recovered among the Malagasy “*Monocentropus*” individuals. The sample from Marojejy, a rainforest site in northeastern Madagascar, was sister to the remaining samples. Samples from the arid-transitional site of Forêt d’Ambre and the arid site of Montagne des Français in the extreme north of the island were similar to each other and to a previously sequenced captive specimen (Lüddecke et al. 2018).

### ﻿Taxonomy

#### ﻿Family Theraphosidae Thorell, 1869

**Subfamily Eumenophorinae Pocock, 1897**

##### ﻿Genus Monocentropus

Taxon classificationAnimaliaAraneaeTheraphosidae

Pocock, 1897

021E22A9-B02A-5CA5-8BF4-610062D29383

Monocentropus Pocock, 1897: 758.

###### Type species.

*Monocentropusbalfouri* Pocock, 1897, by monotypy.

###### Diagnosis.

Among the known genera of Eumenophorinae, *Monocentropus* sensu stricto (defined here as monotypic) resembles *Satyrex* gen. nov. in coxae I and II with dense cluster of plumose/spike setae prolaterally, and only coxa I with a long paddle seta prodorsally (cf. Fig. 10A vs Fig. 10C, D, F; Fig. 12A vs Fig. 12C, D). However, *Monocentropus* sensu stricto can be distinguished by the not-elongated male palp (1.62× longer than the carapace vs 2.23–3.85× longer than the carapace; cf. Fig. 13A vs Fig. 13C–F), a slender embolus narrowly joined to the tegulum (vs a robust embolus broadly joined to the tegulum; cf. Fig. 14A vs Fig. 14C–G), and sac-like, bilobed, broad receptacles that are close to each other (vs well separated receptacles with a narrow stalk and a head that is either solid or bilobed; cf. Fig. 21A vs Fig. 21C–E).

###### Description.

As for the type species.

###### Composition.

Only the type species.

###### Distribution.

Socotra Island (Fig. 25).

##### ﻿ Monocentropus balfouri

Taxon classificationAnimaliaAraneaeTheraphosidae

Pocock, 1897

1B4F97EB-863B-5A0B-BDBD-4914763A93B3

Figs 2A, B , 3A, B , 8A , 9A , 10A , 11A , 12A , 13A , 14A , 15A , 16A , 17A , 18A , 19A , 20A, B , 21A , 22A , 23A

Monocentropus balfouri Pocock, 1897: 759, pl. 41, fig. 1 (♂).
Monocentropus balfouri : Smith 1986: 36, fig. 23 (♂); Smith 1987: 36, fig. 23 (♂); Smith 1990: 42, figs 167–179a (♂); Schmidt 1993: 111, fig. 346 (♂); Schmidt 2001: 13, fig. 1 (♀); Schmidt 2003: 214, figs 644–645 (♂).

###### Type material.

***Holotype*** • ♂ (NHMUK 81-10-6), Yemen: Socotra Govt.: Socotra Island (I.B. Balfour) [not examined].

###### Other material examined.

Yemen: Socotra Govt.: • 3♂ 1♀ (SMNS-Aran-001401, 003509, 004364, 004374), • 1♂ 1♀ (SMF), Socotra Island; • 1♀ (SMNS-Aran-004388), same, 2010; • 1♀ (SMNS-Aran-004390), same, 7.2002 (A. Stirm).

###### Common name.

Socotra Island blue tarantula.

###### Diagnosis.

As for the genus; see also under “*Monocentropus*” *lambertoni*.

###### Description.

**Figure 2.**
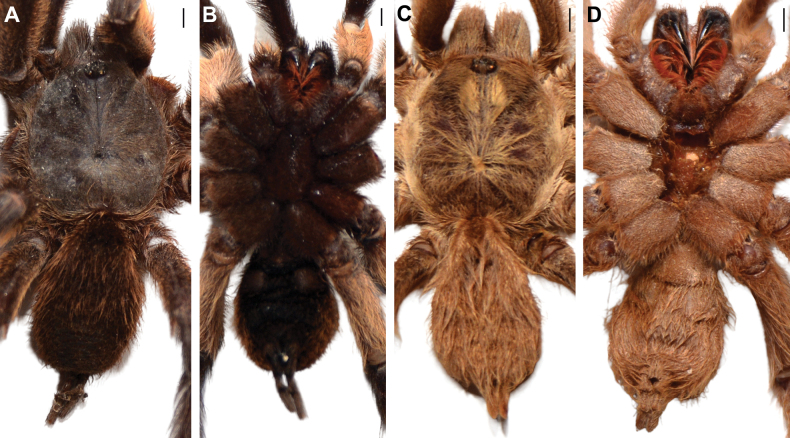
Habitus of males in dorsal (**A, C**) and ventral (**B, D**) views. **A, B.***Monocentropusbalfouri* (SMNS-Aran-003509); **C, D.** “*Monocentropus*” *lambertoni* (SMNS-Aran-004365). Scale bars: 2 mm.

**Male** (SMNS-Aran-003509). Habitus as in Figs 2A, B, 22A. Total length 31.7. Carapace 15.7 long, 14.0 wide. Eye tubercle as in Fig. 8A. Fovea deep and transverse. Stridulatory organs: absent in chelicerae; maxillae with spiniform setae retrolaterally (Fig. 9A); prolateral surface of coxa I dorsally with one large paddle seta, and of coxae I and II with dense cluster of plumose/spike setae (Figs 10A, 12A); prolateral surface of trochanter I dorsally with thick hooked setae (Fig. 10A); prolateral surface of trochanter II dorsally with few thin hooked setae. Cheliceral furrow with eight promarginal teeth and 12 mesobasal denticles. Sternum 7.8 long, 6.3 wide, with two pairs of sigilla. Labium wider than long, with ~165 cuspules. Each maxilla with ~220 cuspules.

Measurements of palp and legs: palp: 25.5 (9.0, 6.0, 8.0, —, 2.5), I: 51.5 (16.0, 7.0, 11.5, 11.0, 6.0), II: 47.0 (12.0, 7.0, 11.0, 10.0, 7.0), III: 46.5 (13.0, 5.5, 9.0, 12.0, 7.0), IV: 55.0 (14.0, 7.0, 12.0, 15.5, 6.5). Full palp as in Fig. 13A; 1.62× longer than carapace. Tibial apophysis as in Fig. 20A, B; small mound with 12 spines. All tarsal and metatarsal scopulae integral. Metatarsal scopulae: I: 75%, II: 70%, III, IV: 55%. Distal tibial spines: I: 2r; II: 2p; III: 1p; IV: 1p1r. Distal ventral metatarsal spines: I: 1m; II, III, IV: 1p1m1r. Paired claws smooth, third claw absent.

Spinnerets: PLS: basal article: 3.05 long, median article: 1.8 long, apical article: 3.0 long. PMS: 1.9 long.

Bulb as in Figs 14A, 15A, 16A, 17A; tegulum oval, ~1.6× wider than long; embolus slender, base relatively narrow and distinct from tegulum, almost straight in lateral view, gently curved in distal/proximal views, gradually tapering towards apex; tip of embolus not expanded, with weak apical (*A*) and pronounced prolateral inferior (*PI*) keels (Figs 18A, 19A).

Colour in life (Fig. 22A): carapace dark metallic blue; legs almost entirely metallic blue, femora with long creamy beige setae; abdomen dark blue, densely covered with creamy beige setae.

**Figure 3.**
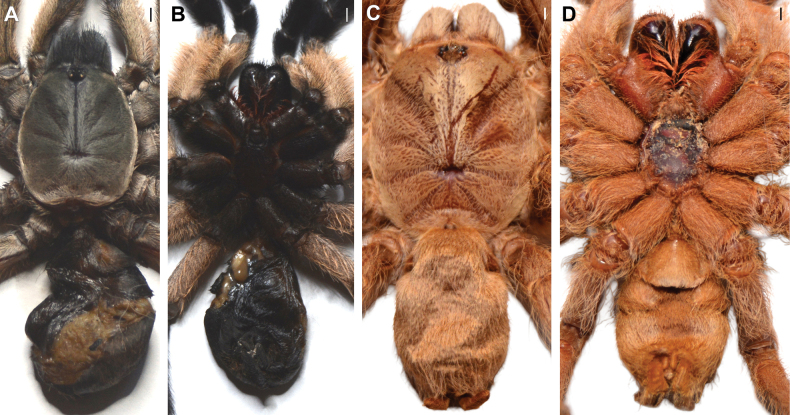
Habitus of females in dorsal (**A, C**) and ventral (**B, D**) views. **A, B.***Monocentropusbalfouri* (SMNS-Aran-004374); **C, D.** “*Monocentropus*” *lambertoni* (SMNS-Aran-004368). Scale bars: 2 mm.

**Female** (SMNS-Aran-004374). Habitus as in Figs 3A, B, 23A. Total length 47.0. Carapace 20.0 long, 16.0 wide. Stridulatory organs as in male. Cheliceral furrow with ten promarginal teeth and ~23 mesobasal denticles of varying sizes. Sternum 10.0 long, 7.9 wide, with two pairs of sigilla. Labium wider than long, with ~140 cuspules. Each maxilla with ~220 cuspules.

Measurements of palp and legs: palp: 30.6 (7.8, 6.5, 8.8, —, 7.5), I: 50.5 (13.0, 7.5, 12.5, 10.0, 7.5), II: 48.0 (13.0, 7.0, 10.5, 9.0, 8.5), III: 48.0 (12.5, 6.5, 9.5, 11.0, 8.5), IV: 56.5 (15.0, 7.0, 12.0, 14.5, 8.0). All tarsal and metatarsal scopulae integral. Metatarsal scopulae: I: 85%, II: 80%, III: 65%, IV: 55%. Distal tibial spines: I: 1r; II, III, IV: 1p,1r. Distal ventral metatarsal spines: I, II: 1m; III: 1p1m; IV: 1p.

Spinnerets: PLS: basal article: 4.75 long, median article: 3.4 long, apical article: 4.45 long. PMS: 2.4 long.

Receptacles as in Fig. 21A; sac-like, bilobed, slightly separated; ental lobe distinctly smaller, digitiform.

Colour in life (Fig. 23A): carapace greyish brown to pale metallic blue; femora creamy beige, more-distal segments blue; abdomen creamy-beige, densely coated with greyish to brownish setae; ventral side of body and legs blackish.

###### Natural history.

A fossorial species that extensively webs the interior and surroundings of its burrow. It typically inhabits rocky montane grasslands with scattered low shrubs and small trees (Fig. 24A).

###### Distribution.

Known only from Socotra Island, Socotra Governorate, Yemen (Fig. 25). None of the material reported in the scientific literature includes precise collection data; the sites marked on the map are based on eight records from the social networking platform iNaturalist (2025).

##### "Monocentropus" lambertoni

Taxon classificationAnimaliaAraneaeTheraphosidae

Fage, 1922

391D8C7F-CA91-5A3C-B9EE-F924303C124C

Figs 2C, D , 3C, D , 8B , 9B, E , 10B , 11B , 12B , 13B , 14B , 15B , 16B , 17B , 18B , 19B , 20C, D , 21B , 22B , 23B

Monocentropus lambertoni Fage, 1922: 365, figs 1–4 (♂♀).
Monocentropus lambertoni : Smith 1990: 43, fig. 179b–e (♂); Schmidt 2003: 214, figs 646–647 (♂).

###### Type material.

***Holotype*** • ♂ (MNHN), Madagascar: further locality and date unknown (M. Lamberton) [examined, courtesy of F. Vol]. ***Paratypes***: • 2♂ 5♀ (MNHN), collected together with the holotype [examined, courtesy of F. Vol].

###### Other material examined.

Madagascar: Diana Reg.: • 2♂ 3♀ (SMNS-Aran-004363, 65–68), 13°23'30.4"S, 48°58'57.5"E, 121 m, 11.2009 (F. Schneider).

###### Common name.

We propose “Madagascan brown tarantula” as a common name.

###### Diagnosis.

“*Monocentropus*” *lambertoni* can be distinguished from *M.balfouri* in having large paddle setae on coxa II (cf. Fig. 12B vs Fig. 12A), a pair of labiosternal mounds (Fig. 9E), and a drastically different coloration pattern (cf. Figs 22B, 23B vs Figs 22A, 23A). The male can be further distinguished from that of *M.balfouri* by the different curvature of the embolus (cf. Fig. 17B vs Fig. 17A) and by the tibial apophysis, which is robust and conical (vs a small mound; cf. Fig. 20C, D vs Fig. 20A, B). The female further differs from that of *M.balfouri* in having both lobes of the receptacle approximately equal in size (vs ectal lobe distinctly larger; cf. Fig. 21B vs Fig. 21A).

###### Description.

**Male** (SMNS-Aran-004365). Habitus as in Figs 2C, D, 22B. Total length 34.7. Carapace 16.4 long, 15.0 wide. Eye tubercle as in Fig. 8B. Stridulatory organs: absent in chelicerae; maxillae with spiniform setae retrolaterally (Fig. 9B); prolateral surface of coxae I and II with dense cluster of plumose/spike setae, and dorsally in coxae I and II with large paddle setae (Figs 10B, 12B); prolateral surface of trochanters I and II dorsally with thick hooked setae (Figs 10B, 12B). Cheliceral furrow with 11 promarginal teeth and 39 mesobasal denticles. Sternum destroyed. Labium wider than long, with ~150 cuspules and outward-curving mounds. Each maxilla with ~270 cuspules.

Measurements of palp and legs: palp: 27.5 (10.0, 5.5, 9.0, —, 3.0), I: 55.0 (14.0, 8.5, 13.0, 12.0, 7.5), II: 50.0 (13.0, 8.0, 11.0, 11.0, 7.0), III: 48.0 (12.5, 6.5, 9.5, 11.0, 8.5), IV: 55.5 (15.0, 6.0, 12.0, 15.0, 7.5). Full palp as in Fig. 13B; 1.67× longer than carapace. Tibial apophysis as in Fig. 20C, D; well-developed and robust, with 23 spines. All tarsal and metatarsal scopulae integral. Metatarsal scopulae: I, II: 85%, III: 75%, IV: 45%. Distal tibial spines: II, III, IV: 1p. Distal ventral metatarsal spines: III: 1p1r; IV: 1p1m1r.

Spinnerets: PLS: basal article: 2.3 long, median article: 1.7 long, apical article: 3.2 long. PMS: 1.8 long.

Bulb as in Figs 14B, 15B, 16B, 17B; tegulum ~1.5× wider than long; embolus gently curved in lateral view, sharply curved in distal/proximal views, slightly tapering only near apex; tip of embolus not expanded, with apical (*A*), prolateral inferior (*PI*) and prolateral superior (*PS*) keels (Figs 18B, 19B).

Colour in life (Fig. 22B): almost uniformly grey or golden brown.

**Female** (SMNS-Aran-004368). Habitus as in Figs 3C, D, 23B. Total length 51.0. Carapace 26.0 long, 23.0 wide. Stridulatory organs as in male. Cheliceral furrow with 13 promarginal teeth and ~ 87 mesobasal denticles. Sternum 12.43 long, 10.42 wide, with 2 pairs of sigilla. Labium wider than long, with ~125 cuspules and outward-curving mounds (Fig. 9E). Each maxilla with ~330 cuspules.

Measurements of palp and legs: palp: 39.5 (12.5, 7.0, 10.0, —, 10.0), I: 64.5 (18.0, 10.5, 14.0, 12.5, 9.5), II: 57.0 (15.0, 8.5, 13.0, 12.5, 8.0), III: 55.5 (14.5, 9.0, 11.0, 14.0, 7.0), IV: 65.0 (17.0, 9.0, 13.0, 18.0, 8.0). All tarsal and metatarsal scopulae integral. Metatarsal scopulae: I, II: 85%, III, IV: 70%. Distal tibial spines: none. Distal ventral metatarsal spines: III, IV: 1p1m1r.

Spinnerets: PLS: basal article: 5.15 long, median article: 4.1 long, apical article: 5.85 long. PMS: 3.05 long.

Receptacles as in Fig. 21B; sac-like, slightly separated, bilobed; lobes of similar shape and size.

Colour in life (Fig. 23B): as in male.

###### Natural history.

Unknown; possibly fossorial.

###### Comments.

“*Monocentropus*” *lambertoni* is here proposed to be considered as incerta sedis, based on both its phylogenetic placement (Fig. 1) and certain morphological features, including the presence of several paddle setae on coxa II (Fig. 12B) and a pair of outward-curving labiosternal mounds (Fig. 9E). The morphological description presented herein is based on specimens with the imprecise locality “Diana Region,” a geographically and environmentally heterogeneous area in northern Madagascar. The phylogenetic tree (Fig. 1) indicates that specimens identified as “*Monocentropus*” *lambertoni*, which likely originate from the same batch collected in the Diana Region, form a clade with individuals from the arid site of Montagne des Français (ca 100–200 m a.s.l.) and the arid-transitional site of Forêt d’Ambre (ca 450 m a.s.l.), both located in Madagascar’s extreme north and within the boundaries of the Diana Region. Given the strong genetic divergences observed among Malagasy populations from (i) Forêt d’Ambre and Montagne des Français, (ii) Nosy Hara, (iii) Ankarana, and (iv) the rainforest site of Marojejy, it is hypothesised that multiple species, some potentially microendemic to restricted ranges, are currently subsumed under this name. Preliminary morphological data suggest that the aforementioned populations share diagnostic characters that distinguish “*Monocentropus*” *lambertoni* from *M.balfouri* and *Satyrex* gen. nov. However, labiosternal mounds of similar prominence to those shown in Fig. 9E have so far only been observed in specimens from Montagne des Français, while adult male specimens from Nosy Hara, as well as adult female specimens from Ankarana and Marojejy, have yet to be examined. The taxonomic placement of these tarantulas, which we currently consider as “*Monocentropus*” *lambertoni* sensu lato, as well as their phylogenetic relationships with the enigmatic, monotypic genus *Encyocrates* Simon, 1892, endemic to Madagascar, will be addressed in a separate paper.

###### Distribution.

Known only from Madagascar (Fig. 25). None of the material reported in the scientific literature includes precise collection data; the sites marked on the map are based on the locality of the specimens examined morphologically in this study, four localities corresponding to samples included in the phylogenetic tree, and one record from the social networking platform iNaturalist (2025).

##### ﻿Genus Satyrex

Taxon classificationAnimaliaAraneaeTheraphosidae

Zamani & von Wirth gen. nov.

B8CF8F7C-927A-5B30-91BE-629AC4A013F3

https://zoobank.org/30456BB7-9BBA-4700-945A-2F75F58C4BC7

###### Type species.

*Satyrexferox* sp. nov.

###### Etymology.

The genus name is a combination of *Satyr*, a part-man, part-beast entity from Greek mythology known for his exceptionally large genitals, and the Latin *rēx*, meaning king. Gender is masculine.

###### Diagnosis.

Among the known genera of Eumenophorinae, *Satyrex* gen. nov. resembles *Monocentropus* in coxae I and II with dense cluster of plumose/spike setae prolaterally, and only coxa I with a long paddle seta prodorsally (cf. Fig. 10C, D, F vs Fig. 10A; Fig. 12C, D vs Fig. 12A). However, species of *Satyrex* gen. nov. can be distinguished by the male palp 2.23–3.85× longer than the carapace (vs 1.62× longer than the carapace; cf. Fig. 13C–F vs Fig. 13A), a robust embolus broadly joined to the tegulum (vs a slender embolus narrowly joined to the tegulum; cf. Fig. 14C–G vs Fig. 14A), and receptacles with a narrow stalk and a head that is either solid or bilobed (vs sac-like, broad, bilobed receptacles; cf. Fig. 21C–E vs Fig. 21A).

###### Description.

Males 18.3–27.0 long, carapace 9.35–13.6 long; females 30.15–49.75 long, carapace 13.15–20.3 long. Fovea deep and transverse. Stridulatory organs: absent in chelicerae; maxillae with spiniform setae retrolaterally (Fig. 9C, D); prolateral surface of coxa I dorsally with one large paddle seta, and of coxae I and II with dense cluster of plumose/spike setae (Figs 10C, D, F, 12C, D); prolateral surface of trochanter I dorsally with thick hooked setae (Fig. 10C, E, F); prolateral surface of trochanter II dorsally with few thin hooked setae (only in species from the Arabian Peninsula; Fig. 12C). Labium wider than long. Sternum with two pairs of sigilla. Tibial apophysis: a low mound with comb of spines, and 1–5 retrolateral spines close to it. Leg formula 1423 or 4123. All tarsal and metatarsal scopulae integral. Paired claws smooth, third claw absent.

Male palp: very elongated (Fig. 13C–F), 2.23–3.85× longer than carapace; subtegulum large and broad; tegulum elongated- or broad oval in lateral view; single retrolateral keel present, starting at base of embolus (Figs 14C–G, 15C–F, 16C–F, 17C–F); embolus robust, broadly joined to tegulum, tip with 1–3 keels (Figs 18C–F, 19C–F).

Receptacles: each with narrow stalk and head, latter either solid or bilobed (Fig. 21C–E).

###### Composition.

Five species: *S.arabicus* sp. nov., *S.ferox* sp. nov., *S.longimanus* comb. nov., *S.somalicus* sp. nov., and *S.speciosus* sp. nov.

###### Distribution.

Somaliland and the southern Arabian Peninsula, including Yemen, Saudi Arabia, and Oman (Fig. 25).

##### ﻿ Satyrex ferox

Taxon classificationAnimaliaAraneaeTheraphosidae

Zamani, von Wirth & Stockmann sp. nov.

48DC92C1-BC4D-58D5-8652-18F38B65879D

https://zoobank.org/DB13F7A8-8595-4214-8B3F-2D60496AA029

Figs 4A, E , 5A, E , 6A, D , 7A, D , 8C , 9C , 10C , 11C , 12C , 13C , 14C , 15C , 16C , 17C , 18C , 19C , 20E, F , 21C , 22C , 23C

###### Type material.

***Holotype*** • ♂ (SMNS-Aran-004389), Yemen: Al Mahrah Govt.: Jabal al Fatk, Hawf, NE Al Ghaydah, 16°39'N, 53°03'E, 1.4.2007 (P. Kabátek). ***Paratypes***: • 1♂ 2♀ (SMNS-Aran-004390, 004391, 004392), collected together with the holotype.

**Figure 4.**
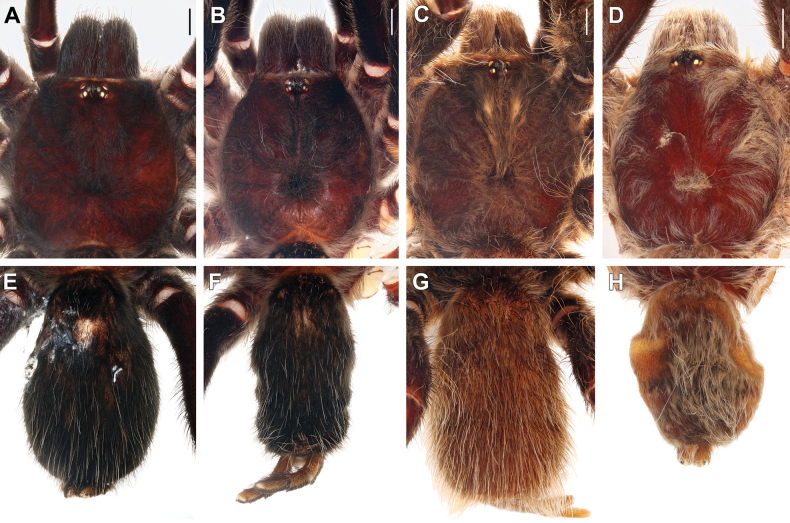
Males of *Satyrex* gen. nov., dorsal view of cephalothorax (**A–D**) and abdomen (**E–G**). **A, E.***S.ferox* sp. nov. (SMNS-Aran-004389); **B, F.***S.arabicus* sp. nov. (SMNS-Aran-004393); **C, G.***S.speciosus* sp. nov. (SMNS-Aran-004395); **D, H.***S.somalicus* sp. nov. (SMNS-Aran-004397). Scale bars: 2 mm.

###### Etymology.

The specific epithet is a Latin adjective meaning ferocious, referring to the aggressive defence behaviour of this species.

**Figure 5.**
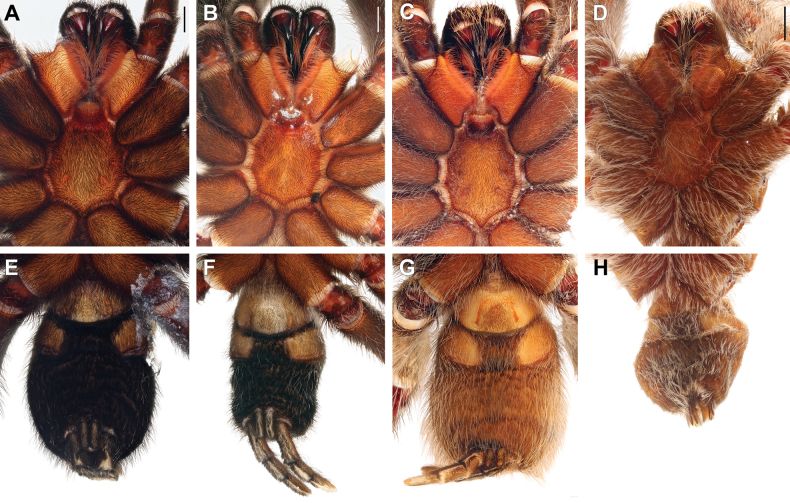
Males of *Satyrex* gen. nov., ventral view of cephalothorax (**A–D**) and abdomen (**E–G**). **A, E.***S.ferox* sp. nov. (SMNS-Aran-004389); **B, F.***S.arabicus* sp. nov. (SMNS-Aran-004393); **C, G.***S.speciosus* sp. nov. (SMNS-Aran-004395); **D, H.***S.somalicus* sp. nov. (SMNS-Aran-004397). Scale bars: 2 mm.

###### Common name.

We propose “Dhofar black tarantula” as a common name.

**Figure 6.**
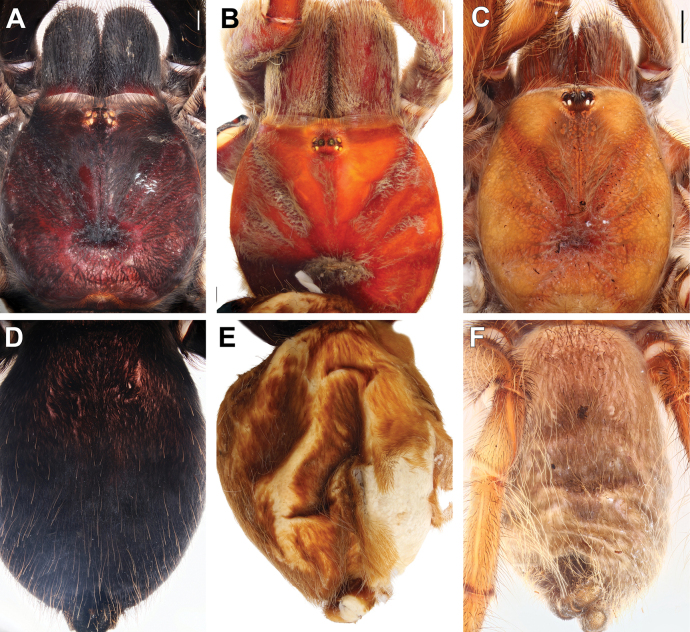
Females of *Satyrex* gen. nov., dorsal view of cephalothorax (**A–C**) and abdomen (**D–F**). **A, D.***S.ferox* sp. nov. (SMNS-Aran-004391); **B, E.***S.longimanus* comb. nov. (NHMUK 03-9-2-30); **C, F.***S.speciosus* sp. nov. (SMNS-Aran-004396). Scale bars: 2 mm.

###### Diagnosis.

The male of *S.ferox* sp. nov. is similar to those of *S.arabicus* sp. nov. and *S.longimanus* comb. nov. in the shape of the bulb. It differs from *S.arabicus* sp. nov. by having a longer, more robust embolus with a gentler, more gradual curvature in lateral view (cf. Fig. 14C vs Fig. 14D). From *S.longimanus* comb. nov., it can be distinguished by the embolus more robust and directed “upward” in lateral view (vs the embolus not as robust and seemingly directed “forward”; cf. Fig. 14C vs Fig. 14G). It can be further differentiated from both species by the longer palp (palp/carapace length ratio: 3.85 vs 3.18 and 2.7, respectively; cf. Fig. 13C vs Fig. 13D). The female differs from other congeners with known females by having receptacles with a longer stalk and a solid, narrow head (vs a short stalk and a broad, bilobed head; cf. Fig. 21C vs Fig. 21D, E).

###### Description.

**Male** (SMNS-Aran-004389). Habitus as in Figs 4A, E, 5A, E, 22C. Total length 27.0. Carapace 12.7 long, 12.0 wide. Eye tubercle as in Fig. 8C. Cheliceral furrow with 12 promarginal teeth and 14 mesobasal denticles. Sternum 6.9 long, 5.6 wide. Labium with ~430 cuspules. Each maxilla with ~320 cuspules.

Measurements of palp and legs: palp: 49.0 (19.4, 7.3, 19.8, —, 2.5), I: 57.15 (17.3, 7.2, 14.35, 12.1, 6.2), II: 48.95 (15.1, 6.15, 11.75, 10.45, 5.5), III: 44.05 (12.7, 5.15, 9.35, 11.15, 5.7), IV: 54.2 (15.0, 6.3, 12.5, 14.15, 6.25). Full palp as in Fig. 13C; 3.85× longer than carapace. Tibial apophysis as in Fig. 20E, F; with 21 spines. Metatarsal scopulae: I: 85%, II: 90%, III: 65%, IV: 50%. Distal tibial spines: I: 2p5r; II: 6p3r; III, IV: 1p1m2r. Distal ventral metatarsal spines: I: 1p1r; II, III, IV: 1p1m1r.

Spinnerets: PLS: basal article: 2.95 long, median article: 1.8 long, apical article: 2.75 long. PMS: 2.2 long.

Bulb as in Figs 14C, 15C, 16C, 17C; tegulum ~1.7× longer than wide, with 2 semicircular outgrowths; embolus gently curved and tapering towards apex, tip with small apical (*A*) keel (Figs 18C, 19C).

Colour in life (Fig. 22C): overall body and legs dark greyish blue to almost black.

**Figure 7.**
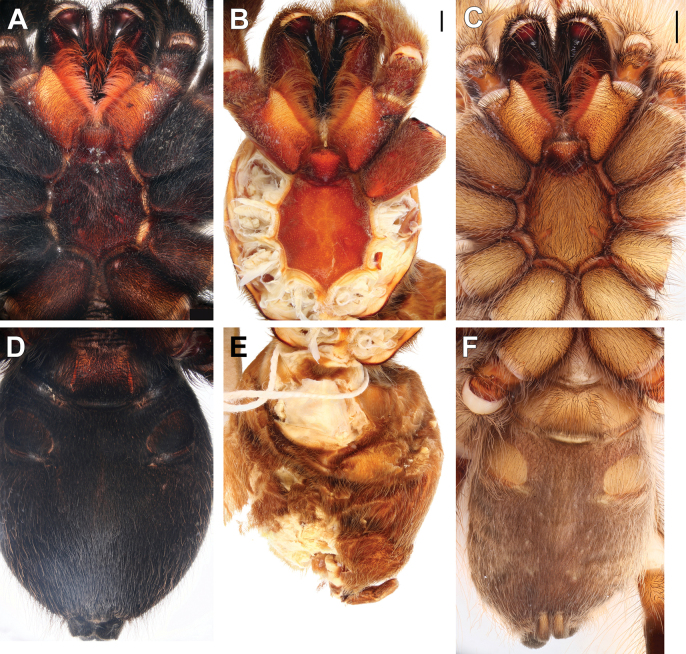
Females of *Satyrex* gen. nov., ventral view of cephalothorax (**A–C**) and abdomen (**D–F**). **A, D.***S.ferox* sp. nov. (SMNS-Aran-004391); **B, E.***S.longimanus* comb. nov. (NHMUK 03-9-2-30); **C, F.***S.speciosus* sp. nov. (SMNS-Aran-004396). Scale bars: 2 mm.

**Female** (SMNS-Aran-004391). Habitus as in Figs 6A, D, 7A, D, 23C. Total length 49.75. Carapace 20.3 long, 20.35 wide. Stridulatory organs as in Figs 9C, 10C, 11C, 12C. Cheliceral furrow with 12 promarginal teeth and nine mesobasal denticles. Sternum 9.5 long, 9.6 wide. Labium with ~485 cuspules. Each maxilla with ~400 cuspules.

**Figure 8.**
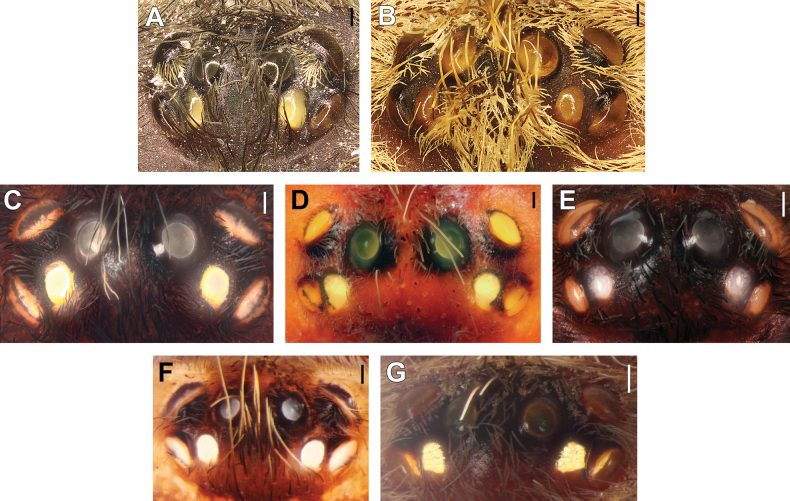
Eye tubercles, males (**A–C, E, G**) and females (**D, F**). **A.***Monocentropusbalfouri* (SMNS-Aran-003509); **B.** “*Monocentropus*” *lambertoni* (SMNS-Aran-004365); **C.***Satyrexferox* sp. nov. (SMNS-Aran-004389); **D.***S.longimanus* comb. nov. (NHMUK 03-9-2-30); **E.***S.arabicus* sp. nov. (SMNS-Aran-004393); **F.***S.speciosus* sp. nov. (SMNS-Aran-004396); **G.***S.somalicus* sp. nov. (SMNS-Aran-004397). Scale bars: 0.2 mm.

**Figure 9.**
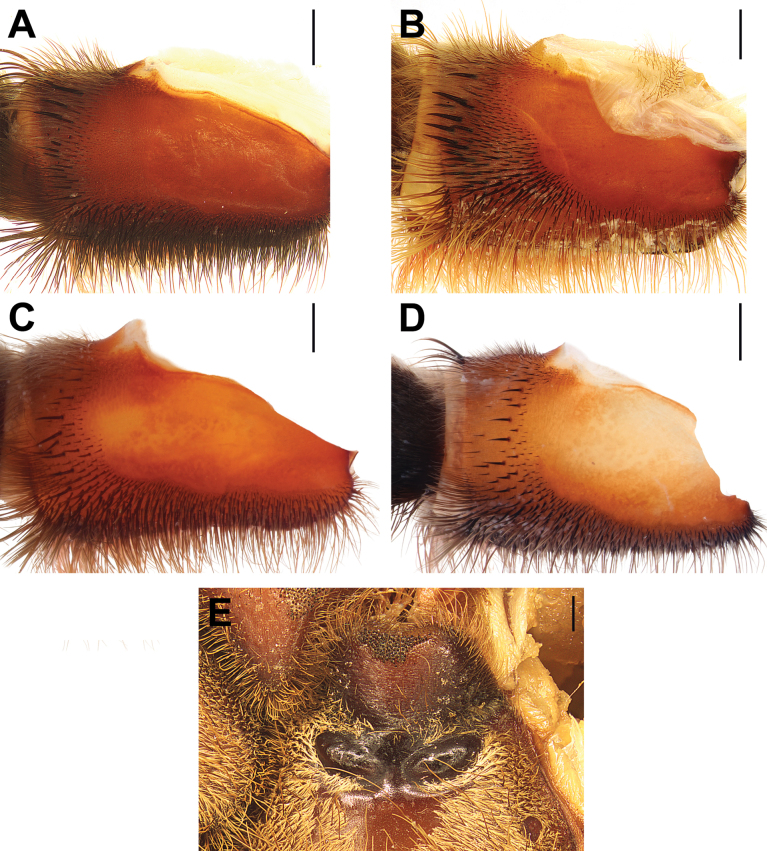
Maxillae, retrolateral view (**A–D**) and labiosternal mounds (**E**), males (**A, B**) and females (**C–E**). **A.***Monocentropusbalfouri* (SMNS-Aran-003509); **B, E.** “*Monocentropus*” *lambertoni* (SMNS-Aran-004368); **C.***Satyrexferox* sp. nov. (SMNS-Aran-004392); **D.***S.speciosus* sp. nov. (SMNS-Aran-004396). Scale bars: 1 mm.

**Figure 10.**
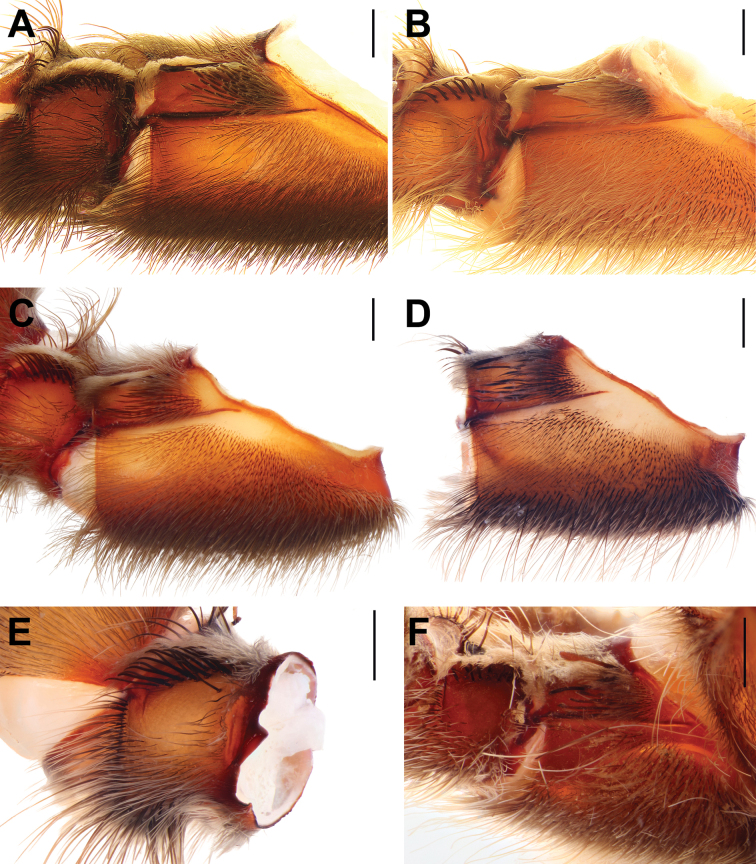
Coxae and trochanters I, prolateral view, males (**A, B, F**) and females (**C–E**). **A.***Monocentropusbalfouri* (SMNS-Aran-003509); **B.** “*Monocentropus*” *lambertoni* (SMNS-Aran-004365); **C.***Satyrexferox* sp. nov. (SMNS-Aran-004392); **D, E.***S.speciosus* sp. nov. (coxa and trochanter separated; SMNS-Aran-004396); **F.***S.somalicus* sp. nov. (SMNS-Aran-004397). Scale bars: 1 mm.

**Figure 11.**
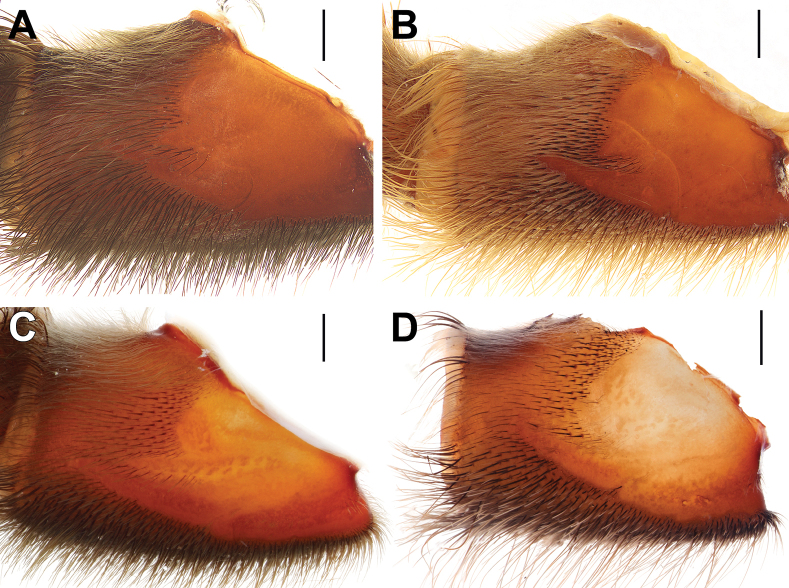
Coxae I, retrolateral view, males (**A, B**) and females (**C, D**). **A.***Monocentropusbalfouri* (SMNS-Aran-003509); **B.** “*Monocentropus*” *lambertoni* (SMNS-Aran-004365); **C.***Satyrexferox* sp. nov. (SMNS-Aran-004392); **D.***S.speciosus* sp. nov. (SMNS-Aran-004396). Scale bars: 1 mm.

**Figure 12.**
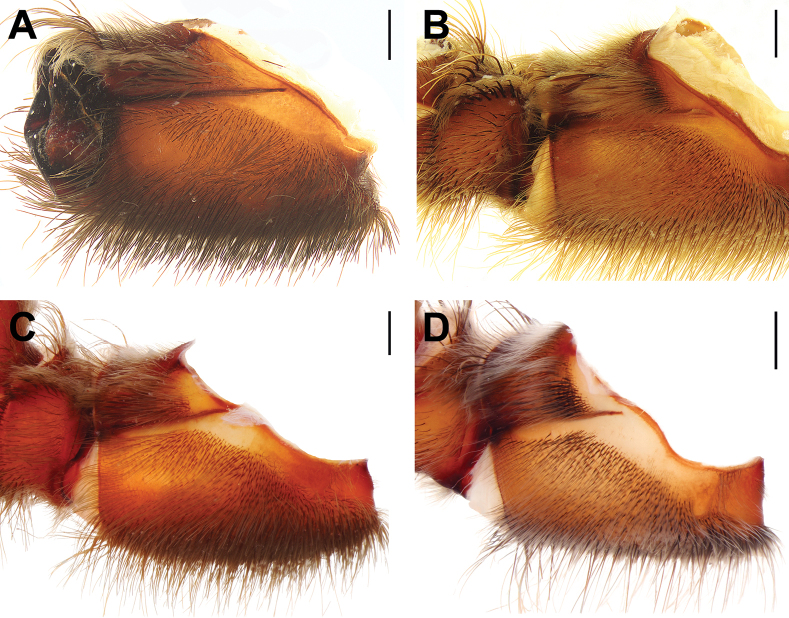
Coxae and trochanters II, prolateral view, males (**A, B**) and females (**C, D**). **A.***Monocentropusbalfouri* (coxa only; SMNS-Aran-003509); **B.** “*Monocentropus*” *lambertoni* (SMNS-Aran-004365); **C.***Satyrexferox* sp. nov. (SMNS-Aran-004392); **D.***S.speciosus* sp. nov. (SMNS-Aran-004396). Scale bars: 1 mm.

**Figure 13.**
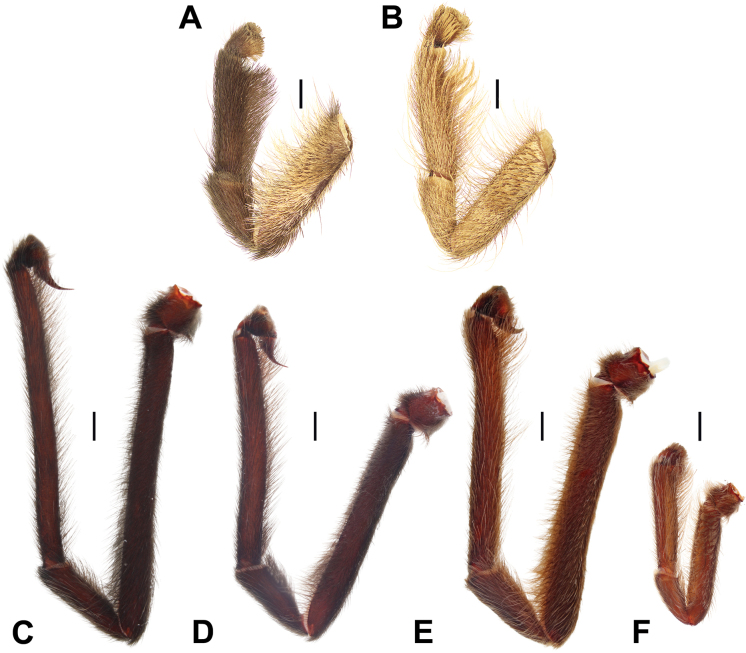
Full male palps, prolateral view. **A.***Monocentropusbalfouri* (SMNS-Aran-003509); **B.** “*Monocentropus*” *lambertoni* (SMNS-Aran-004365); **C.***Satyrexferox* sp. nov. (SMNS-Aran-004389); **D.***S.arabicus* sp. nov. (SMNS-Aran-004393); **E.***S.speciosus* sp. nov. (SMNS-Aran-004395); **F.***S.somalicus* sp. nov. (SMNS-Aran-004397). Scale bars: 2 mm.

**Figure 14.**
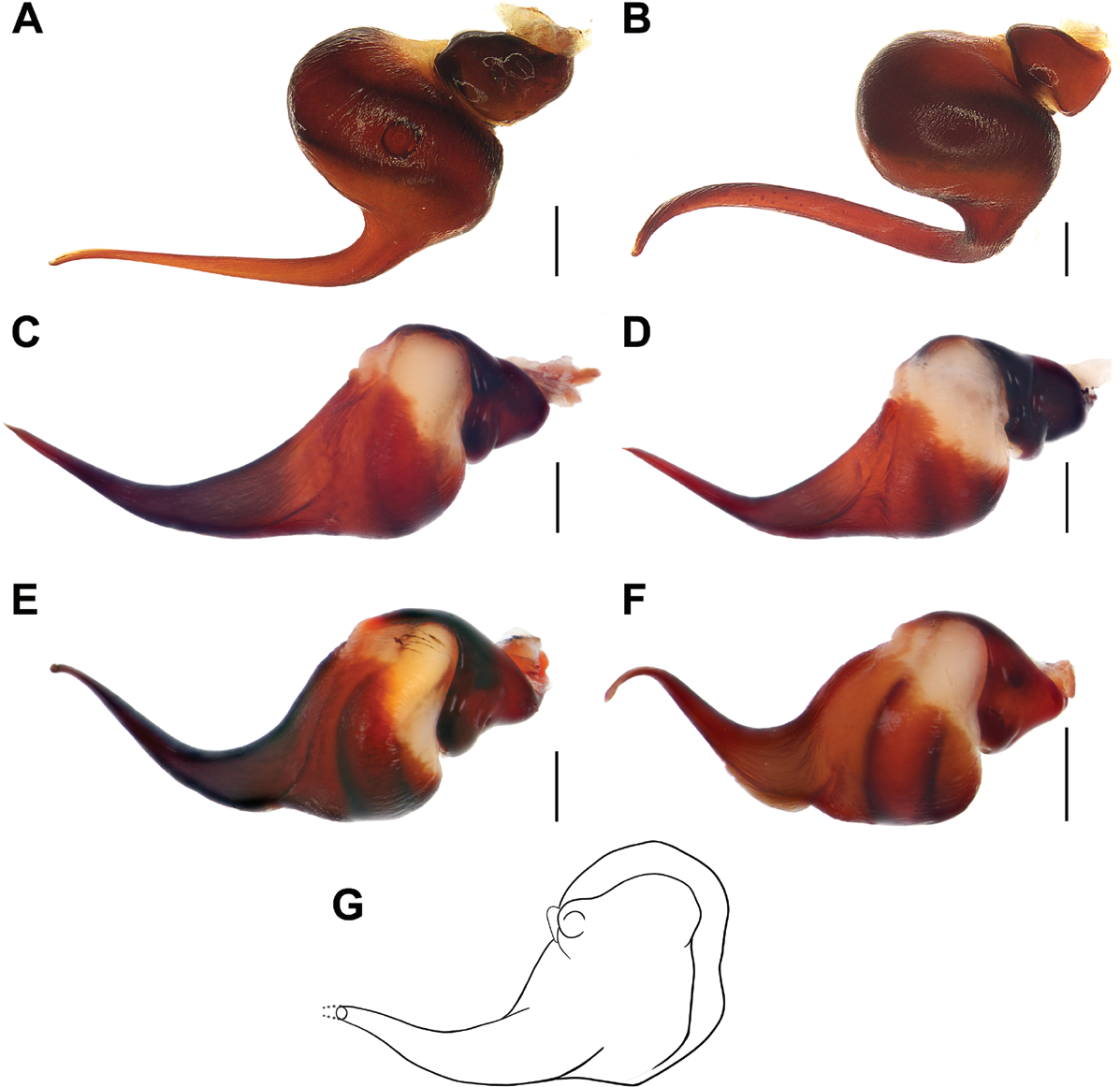
Bulbs, retrolateral view. **A.***Monocentropusbalfouri* (SMNS-Aran-003509); **B.** “*Monocentropus*” *lambertoni* (SMNS-Aran-004365); **C.***Satyrexferox* sp. nov. (SMNS-Aran-004389); **D.***S.arabicus* sp. nov. (SMNS-Aran-004393); **E.***S.speciosus* sp. nov. (SMNS-Aran-004395); **F.***S.somalicus* sp. nov. (SMNS-Aran-004397); **G.***S.longimanus* comb. nov. (NHMUK 03-9-2-29), by Mahla Pourcheraghi, after Smith (1990). Scale bars: 0.5 mm.

Measurements of palp and legs: palp: 38.45 (12.15, 8.1, 10.0, —, 8.2), I: 58.4 (16.35, 11.0, 12.95, 11.0, 7.1), II: 53.55 (15.1, 9.0, 11.9, 10.75, 6.8), III: 50.15 (14.15, 8.55, 9.5, 11.1, 6.85), IV: 58.05 (16.45, 9.25, 12.3, 12.65, 7.4). Metatarsal scopulae: I, II: 90%, III: 85%, IV: 65%. Distal tibial spines: I, II, III, IV: 1p1m1r. Distal ventral metatarsal spines: I, II, III, IV: 1p1m1r.

Spinnerets: PLS: basal article: 3.15 long, median article: 2.4 long, apical article: 3.75 long. PMS: 2.95 long.

Receptacles as in Fig. 21C; stalk relatively long and narrow; head poorly defined, solid.

Colour in life (Fig. 23C): as in male.

###### Natural history.

A fossorial species that constructs its burrow at the base of shrubs or between rocks (Fig. 24C). Both sexes are highly defensive and, at the slightest provocation, assume a threat posture and produce loud, frequent stridulation. The area in which live specimens were observed and photographed is a well-vegetated, rocky wadi on the southern slopes of the Dhofar mountain range in Oman (Fig. 24B). The surrounding area is covered in primary forest, and the substrate comprises clay soil interspersed with numerous rocks and boulders.

###### Distribution.

Known from the type locality in the Al Mahrah Governorate, southeastern Yemen (light green hexagon on the map; Fig. 25), as well as from four additional records in the Dhofar Governorate, southwestern Oman (dark green hexagons), based on observations from the social networking platform iNaturalist (2025).

##### ﻿ Satyrex arabicus

Taxon classificationAnimaliaAraneaeTheraphosidae

Zamani & von Wirth sp. nov.

B5D4C58A-5CEC-58CF-8853-9D4E7E2454F2

https://zoobank.org/E0483ACD-A17C-47DB-B19C-B1D930906FEC

Figs 4B, F , 5B, F , 8E , 13D , 14D , 15D , 16D , 17D , 18D , 19D , 20G, H , 22D , 23D

###### Type material.

***Holotype*** • ♂ (SMNS-Aran-004393), Saudi Arabia: Jazan Prov.: Faifa Mountains, 17°15'N, 43°06'E, 22.7.2024 (I.M. Fageeh). ***Paratype***: • 1♂ (SMNS-Aran-004394), same locality and collector, 15.10.2024.

###### Etymology.

The specific epithet refers to the distribution of the species in Saudi Arabia.

###### Common name.

We propose “Arabian black tarantula” as a common name.

###### Diagnosis.

The male of *S.arabicus* sp. nov. is similar to those of *S.ferox* sp. nov. and *S.longimanus* comb. nov. in the shape of the bulb. It differs from *S.ferox* sp. nov. by having a shorter, less robust embolus, with a sharper, more pronounced curvature, giving it a more acute angle in lateral view (cf. Fig. 14D vs Fig. 14C), and a shorter palp (palp/carapace length ratio: 3.18 vs 3.85; cf. Fig. 13D vs Fig. 13C). From *S.longimanus* comb. nov., it can be distinguished by the embolus directed “upward” (vs the embolus apparently directed “forward”; cf. Fig. 14D vs Fig. 14G), and a longer palp (palp/carapace length ratio: 3.18 vs 2.7).

###### Description.

**Male** (SMNS-Aran-004393). Habitus as in Figs 4B, F, 5B, F, 22D. Total length 25.5. Carapace 12.0 long, 11.6 wide. Eye tubercle as in Fig. 8E. Cheliceral furrow with 11 promarginal teeth and 11 mesobasal denticles. Sternum 6.0 long, 5.3 wide. Labium with ~240 cuspules. Each maxilla with ~210 cuspules.

Measurements of palp and legs: palp: 38.25 (14.55, 6.15, 15.3, —, 2.25), I: 53.85 (15.75, 7.6, 12.85, 11.5, 6.15), II: 47.85 (14.45, 5.5, 11.2, 10.9, 5.8), III: 44.05 (12.05, 5.8, 9.15, 11.05, 6.0), IV: 54.2 (14.65, 6.0, 12.25, 14.7, 6.6). Full palp as in Fig. 13D; 3.18× longer than carapace. Tibial apophysis as in Fig. 20G, H; with 17 spines. Metatarsal scopulae: I, II: 80%, III: 60%, IV: 35%. Distal tibial spines: I: 1p3r; II: 2p2r; III, IV: 2p1m1r. Distal ventral metatarsal spines: I: 2p1m1r; II, III, IV: 1p1m1r.

Spinnerets: PLS: basal article: 2.8 long, median article: 1.75 long, apical article: 2.6 long. PMS: 2.15 long.

**Figure 15.**
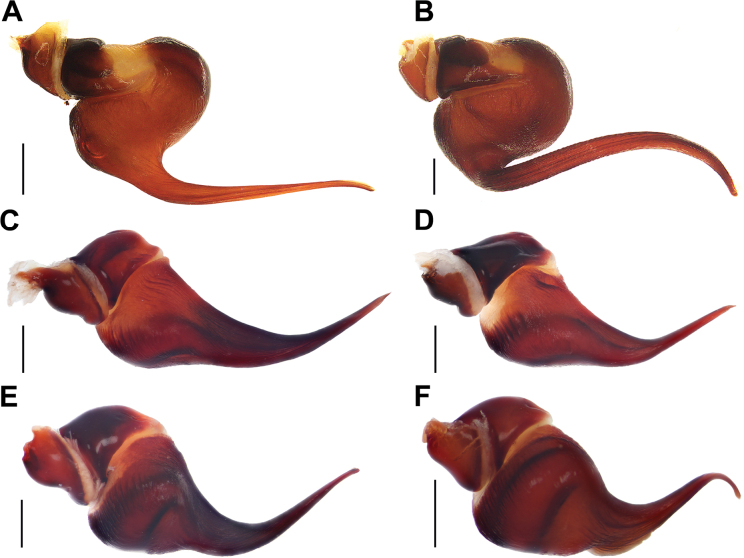
Bulbs, prolateral view. **A.***Monocentropusbalfouri* (SMNS-Aran-003509); **B.** “*Monocentropus*” *lambertoni* (SMNS-Aran-004365); **C.***Satyrexferox* sp. nov. (SMNS-Aran-004389); **D.***S.arabicus* sp. nov. (SMNS-Aran-004393); **E.***S.speciosus* sp. nov. (SMNS-Aran-004395); **F.***S.somalicus* sp. nov. (SMNS-Aran-004397). Scale bars: 0.5 mm.

**Figure 16.**
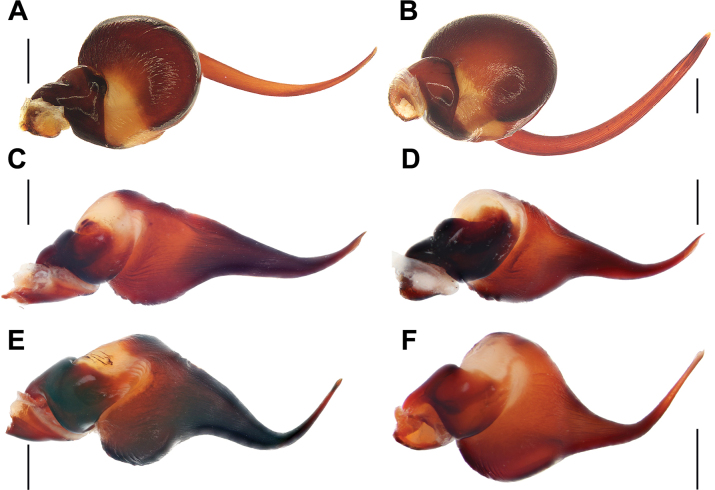
Bulbs, distal view. **A.***Monocentropusbalfouri* (SMNS-Aran-003509); **B.** “*Monocentropus*” *lambertoni* (SMNS-Aran-004365); **C.***Satyrexferox* sp. nov. (SMNS-Aran-004389); **D.***S.arabicus* sp. nov. (SMNS-Aran-004393); **E.***S.speciosus* sp. nov. (SMNS-Aran-004395); **F.***S.somalicus* sp. nov. (SMNS-Aran-004397). Scale bars: 0.5 mm.

**Figure 17.**
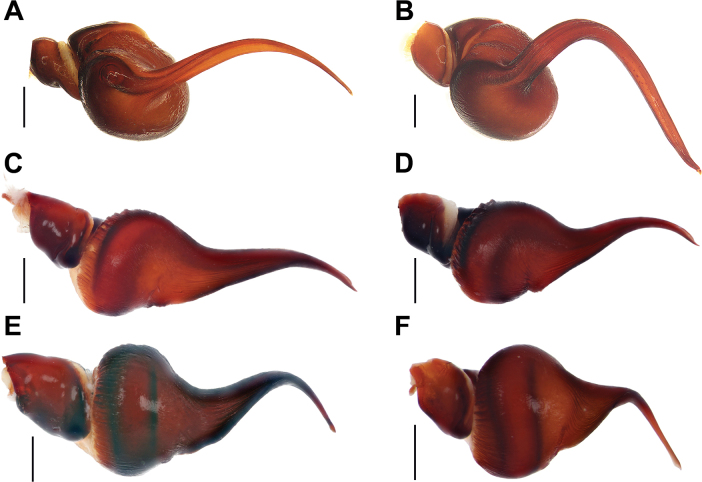
Bulbs, proximal view. **A.***Monocentropusbalfouri* (SMNS-Aran-003509); **B.** “*Monocentropus*” *lambertoni* (SMNS-Aran-004365); **C.***Satyrexferox* sp. nov. (SMNS-Aran-004389); **D.***S.arabicus* sp. nov. (SMNS-Aran-004393); **E.***S.speciosus* sp. nov. (SMNS-Aran-004395); **F.***S.somalicus* sp. nov. (SMNS-Aran-004397). Scale bars: 0.5 mm.

**Figure 18.**
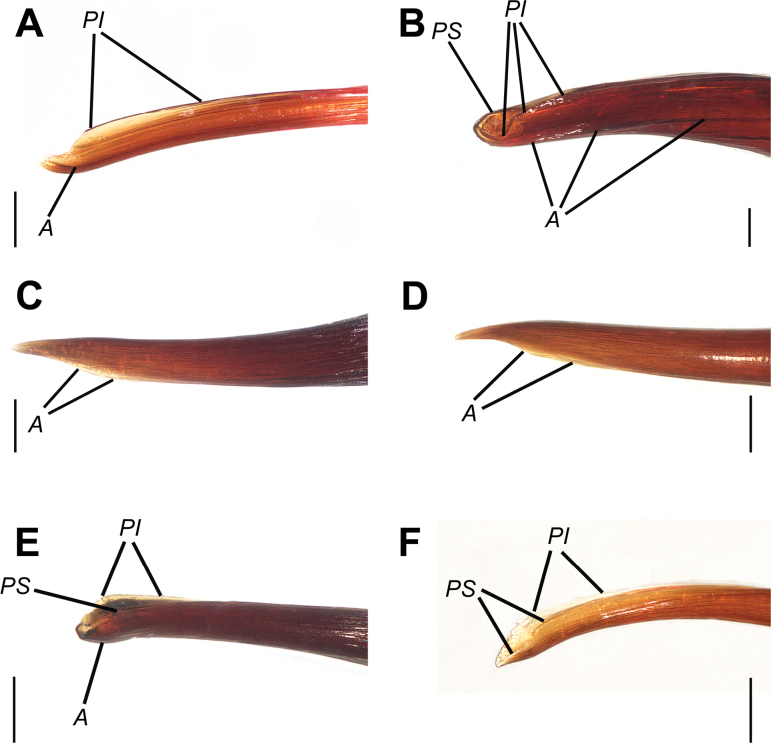
Tips of the emboli, retrolateral view. **A.***Monocentropusbalfouri* (SMNS-Aran-003509); **B.** “*Monocentropus*” *lambertoni* (SMNS-Aran-004365); **C.***Satyrexferox* sp. nov. (SMNS-Aran-004389); **D.***S.arabicus* sp. nov. (SMNS-Aran-004393); **E.***S.speciosus* sp. nov. (SMNS-Aran-004395); **F.***S.somalicus* sp. nov. (SMNS-Aran-004397). Abbreviations: A – apical keel, PI – prolateral inferior keel, PS – prolateral superior keel. Scale bars: 0.1 mm.

**Figure 19.**
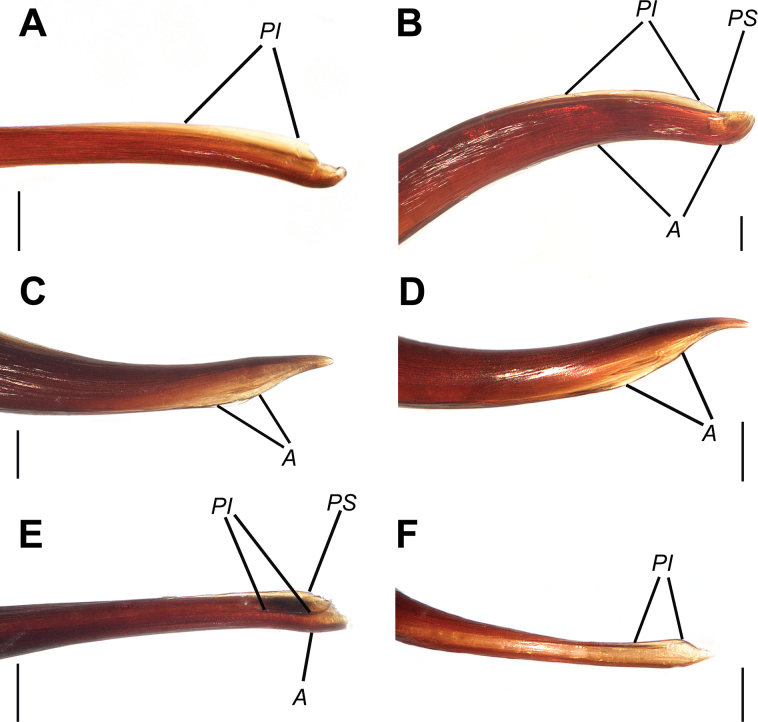
Tips of the emboli, prolateral view. **A.***Monocentropusbalfouri* (SMNS-Aran-003509); **B.** “*Monocentropus*” *lambertoni* (SMNS-Aran-004365); **C.***Satyrexferox* sp. nov. (SMNS-Aran-004389); **D.***S.arabicus* sp. nov. (SMNS-Aran-004393); **E.***S.speciosus* sp. nov. (SMNS-Aran-004395); **F.***S.somalicus* sp. nov. (SMNS-Aran-004397). Abbreviations: A – apical keel, PI – prolateral inferior keel, PS – prolateral superior keel. Scale bars: 0.1 mm.

Bulb as in Figs 14D, 15D, 16D, 17D; tegulum ~1.75× longer than wide, with three semicircular outgrowths; embolus sharply curved and tapering towards apex; embolus with small apical (*A*) keel (Figs 18D, 19D).

**Figure 20.**
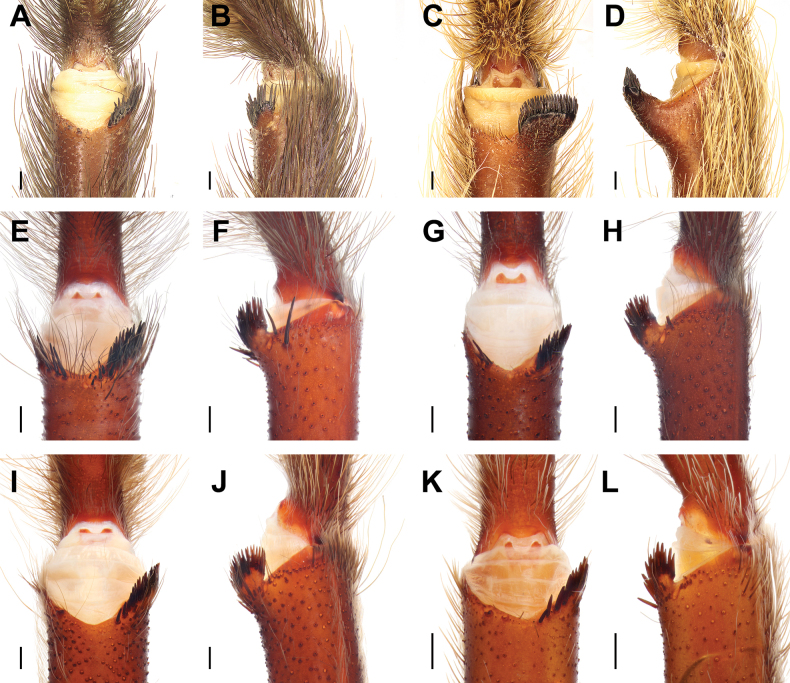
Tibial apophyses, ventral (**A, C, E, G, I, K**) and prolateral (**B, D, F, H, J, L**) views. **A, B.***Monocentropusbalfouri* (SMNS-Aran-003509); **C, D.** “*Monocentropus*” *lambertoni* (SMNS-Aran-004365); **E, F.***Satyrexferox* sp. nov. (SMNS-Aran-004389); **G, H.***S.arabicus* sp. nov. (SMNS-Aran-004393); **I, J.***S.speciosus* sp. nov. (SMNS-Aran-004395); **K, L.***S.somalicus* sp. nov. (SMNS-Aran-004397). Scale bars: 0.5 mm.

Colour in life (Fig. 22D): similar to *S.ferox* sp. nov.

**Female.** Live habitus as in Fig. 23D; colour in life similar to *S.ferox* sp. nov. No preserved specimens available for description.

###### Natural history.

The habitat at the type locality is a humid montane environment with dense vegetation, rocky outcrops, and tall grasses (Fig. 24D). Lifestyle and behaviour similar to *S.ferox* sp. nov.

###### Comments.

There is a possibility that this nomen may become a junior synonym of *S.longimanus* comb. nov. in the future. Unfortunately, we were unable to study a female specimen from Saudi Arabia, and the male holotype of *S.longimanus* comb. nov. appears to have been lost, with only a schematic illustration of its bulb (with embolus broken) available in the literature. The collection sites of the Saudi Arabian and Yemeni specimens are both located in the Sarawat Mountain range, approximately 370 km apart in aerial distance.

###### Distribution.

Known only from the type locality in the Jazan Province, southern Saudi Arabia (Fig. 25).

##### ﻿ Satyrex longimanus

Taxon classificationAnimaliaAraneaeTheraphosidae

(Pocock, 1903) comb. nov.

808B72D1-E8E1-5FEC-8DB2-BF6D68203C9A

**Figure 21.**
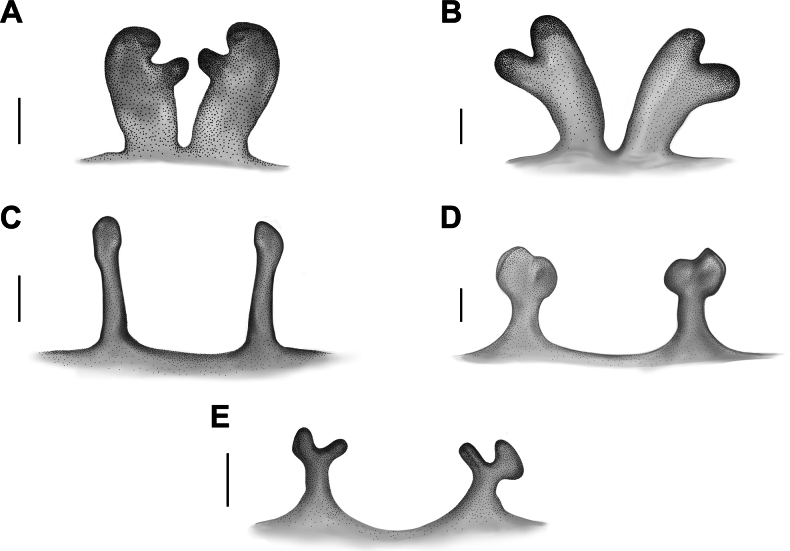
Receptacles, dorsal view. **A.***Monocentropusbalfouri* (SMNS-Aran-004374); **B.** “*Monocentropus*” *lambertoni* (SMNS-Aran-004368); **C.***Satyrexferox* sp. nov. (SMNS-Aran-004391); **D.***S.longimanus* comb. nov. (NHMUK 03-9-2-30); **E.***S.speciosus* sp. nov. (SMNS-Aran-004396). Illustrations by Mahla Pourcheraghi. Scale bars: 0.5 mm.

Figs 6B, E , 7B, E , 8D , 14G , 21D

Monocentropus longimanus Pocock, 1903: 219 (♂).
Monocentropus longimanus : Smith 1990: 43, figs 180–187 (♂); Schmidt 2003: 214, figs 648–649 (♂).

###### Type material.

***Holotype*** • ♂ (NHMUK 03-9-2-29), Yemen: Dhale Govt.: Dthala [= Dhale], El Kubar [= Al Kubar] (G.W. Berry) [not examined].

###### Other material examined.

Yemen: Dhale Govt.: • 1♀ (NHMUK 03-9-2-30), collected together with the holotype [examined].

###### Common name.

We propose “Yemeni black tarantula” as a common name.

###### Diagnosis.

The male of *S.longimanus* comb. nov. is similar to those of *S.ferox* sp. nov. and *S.arabicus* sp. nov. in the shape of the bulb. It differs from both species by the distal part of the embolus seemingly pointing “forward” (vs the distal part pointing “upward”; cf. Fig. 14G vs Fig. 14C, D), and by the shorter palp (palp/carapace length ratio: 2.7 vs 3.85 and 3.18, respectively). From *S.ferox* sp. nov., it can be further distinguished by the less robust embolus (cf. Fig. 14G vs Fig. 14C). The female is most similar to that of *S.speciosus* sp. nov. in the shape of the receptacles, but it can be distinguished by its poorly defined lobes, which are of subequal size and shape (vs well-defined lobes, with the outer lobe larger than the inner one; cf. Fig. 21D vs Fig. 21E).

**Figure 22.**
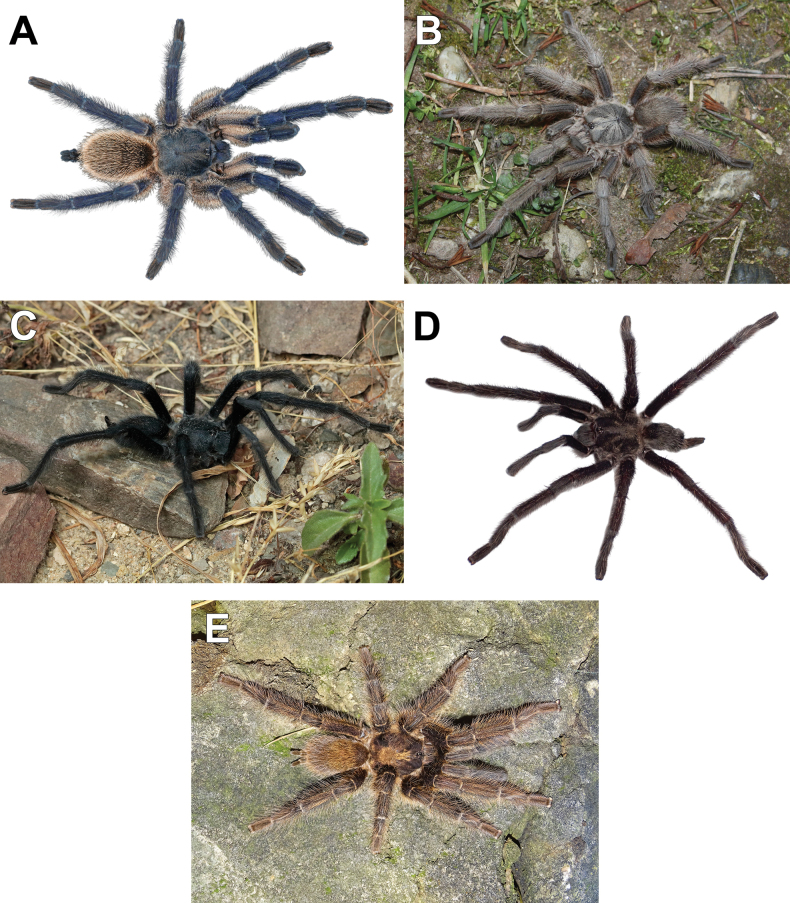
Live habitus of males. **A.***Monocentropusbalfouri*; **B.** “*Monocentropus*” *lambertoni*; **C.***Satyrexferox* sp. nov.; **D.***S.arabicus* sp. nov. (SMNS-Aran-004393); **E.***S.speciosus* sp. nov. (SMNS-Aran-004395). Photos by PF (**A**), Frank Schneider (**B**), Bobby Bok (**C**), Ibrahim Mohssin Fageeh (**D**), and PJ (**E**).

###### Description.

**Male** (holotype, after Pocock (1903) and Smith (1990)). Total length 24.0. Carapace 10.0 long.

Measurements of palp and legs: palp: 27, I: 41, II: 37, III: 35, IV: 44. Palp 2.7× longer than carapace. Tibial apophysis with eight spines. Distal tibial spines: I, II: 2.

Bulb as in Fig. 14G; tegulum ~1.4× longer than wide, with one semicircular outgrowth; embolus broken, gently curved and tapering.

Colour in life: unknown.

**Female** (NHMUK 03-9-2-30). Habitus as in Figs 6B, E, 7B, E. Total length 40.45. Carapace 20.25 long, 18.5 wide. Eye tubercle as in Fig. 8D. Cheliceral furrow with 14 promarginal teeth and 14 mesobasal denticles. Sternum 9.2 long, 8.95 wide. Labium with ~400 cuspules. Each maxilla with ~200 cuspules.

Measurements of palp and legs: palp: 36.6 (12.0, 7.15, 9.65, —, 7.8), I: 53.85 (16.5, 9.5, 11.8, 10.3, 5.75), II: 50.55 (15.0, 9.2, 10.5, 10.2, 5.65), III: 48.5 (13.85, 7.95, 9.0, 10.8, 6.9), IV: 60.1 (17.0, 9.5, 11.9, 14.45, 7.25). Metatarsal scopulae: I: 85%, II: 80%, III: 65%, IV: 50%. Distal tibial spines: I, II, III, IV: 1p1m1r. Distal ventral metatarsal spines: I, II, III, IV: 1p1m1r.

Spinnerets: PLS: basal article: 4.85 long, median article: 4.35 long, apical article: 4.1 long. PMS seemingly detached.

Receptacles as in Fig. 21D; stalk short and narrow; apical lobes poorly defined, outer lobe slightly larger than inner lobe.

Colour in life: unknown.

###### Comments.

The holotype male could not be located at the NHMUK and is presumed to have been lost. The examined female was apparently intended to be designated as a paratype, but this was not mentioned in the original description by Pocock (1903). Although it was later examined and partially described by Smith (1990), no illustrations of its receptacles were provided.

###### Natural history.

Unknown; lifestyle and behaviour likely similar to *S.ferox* sp. nov.

###### Distribution.

Known only from the type locality in the Dhale Governorate, southwestern Yemen (Fig. 25).

##### ﻿ Satyrex speciosus

Taxon classificationAnimaliaAraneaeTheraphosidae

Zamani, von Wirth & Just sp. nov.

EDC72260-D44A-5C2D-9881-34C74912E8AF

https://zoobank.org/315E8134-3543-4834-814E-96F7A1117765

Figs 4C, G , 5C, G , 6C, F , 7C, F , 8F , 9D , 10D, E , 11D , 12D , 13E , 14E , 15E , 16E , 17E , 18E , 19E , 20I, J , 21E , 22E , 23E

###### Type material.

***Holotype*** • ♂ (SMNS-Aran-004395), Somaliland: Sanaag Reg.: Daallo, 10°48'N, 47°19'E, 4.9.2017 (P. Just). ***Paratype***: • 1♀ (SMNS-Aran-004396), collected together with the holotype.

###### Etymology.

The specific epithet is a Latin adjective meaning beautiful.

###### Common name.

We propose “Somali blonde tarantula” as a common name.

###### Diagnosis.

The male of *S.speciosus* sp. nov. resembles that of *S.somalicus* sp. nov. in the shape of the bulb. It differs in its larger size (carapace length 13.6 vs 9.35), by the longer palp (palp/carapace length ratio: 3.13 vs 2.23; cf. Fig. 13E vs Fig. 13F), the tegulum longer than wide (vs almost as long as wide), the retrolateral keel of the bulb poorly developed (vs well-developed), and the different curvature of the embolus (cf. Fig. 14E vs Fig. 14F). Among congeners with known females, the female of *S.speciosus* sp. nov. most closely resembles that of *S.longimanus* comb. nov. in the shape of the receptacles, but can be distinguished by its well-defined lobes, with the outer lobe larger than the inner one (vs poorly defined, subequal lobes; cf. Fig. 21E vs Fig. 21D).

###### Description.

**Male** (SMNS-Aran-004395). Habitus as in Figs 4C, G, 5C, G, 22E. Total length 27.0. Carapace 13.6 long, 12.0 wide. Cheliceral furrow with 11 promarginal teeth and ten mesobasal denticles. Sternum 6.4 long, 5.25 wide. Labium with ~150 cuspules. Each maxilla with ~210 cuspules.

Measurements of palp and legs: palp: 42.7 (16.05, 7.1, 16.95, —, 2.6), I: 49.9 (14.9, 7.35, 10.85, 10.65, 6.15), II: 42.55 (12.85, 6.05, 8.5, 9.7, 5.45), III: 39.25 (11.2, 5.7, 6.6, 10.25, 5.5), IV: 48.2 (13.3, 6.1, 9.65, 13.2, 5.95). Full palp as in Fig. 13E; 3.13× longer than carapace. Tibial apophysis as in Fig. 20I, J; with 24 spines. Metatarsal scopulae: I: 90%, II: 95%, III: 80%, IV: 75%. Distal tibial spines: I: 1r; II, III, IV: 1p1r. Distal ventral metatarsal spines: I, II, III, IV: 1p1m1r.

Spinnerets: PLS: basal article: 2.3 long, median article: 2.0 long, apical article: 2.4 long. PMS: 1.7 long.

Bulb as in Figs 14E, 15E, 16E, 17E; tegulum ~1.75× longer than wide; embolus sharply curved and tapering towards apex, tip with apical (*A*), prolateral inferior (*PI*), and prolateral superior (*PS*) keels (Figs 18E, 19E).

Colour in life (Fig. 22E): overall body and legs dark golden brown.

**Figure 23.**
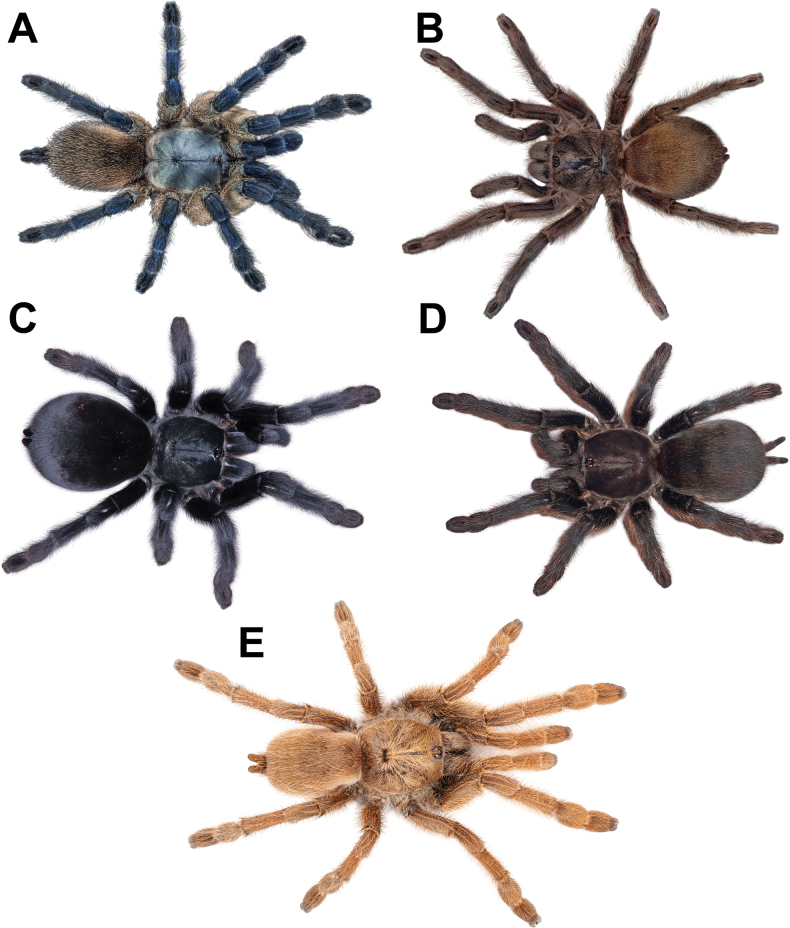
Live habitus of females. **A.***Monocentropusbalfouri*; **B.** “*Monocentropus*” *lambertoni*; **C.***Satyrexferox* sp. nov.; **D.***S.arabicus* sp. nov.; **E.***S.speciosus* sp. nov. Photos by PF (**A, B, E**), MS (**C**), and Ibrahim Mohssin Fageeh (**D**).

**Female** (SMNS-Aran-004396). Habitus as in Figs 6C, F, 7C, F, 23E. Total length 30.15. Carapace 13.15 long, 11.45 wide. Eye tubercle as in Fig. 8F. Stridulatory organs as in Figs 9D, 10D, E, 11D, 12D. Cheliceral furrow with 11 promarginal teeth and ~35 mesobasal denticles of varying size. Sternum 6.3 long, 5.3 wide. Labium with ~160 cuspules. Each maxilla with ~180 cuspules.

Measurements of palp and legs: palp: 24.25 (8.15, 4.85, 5.85, —, 5.4), I: 36.7 (10.95, 6.6, 7.8, 6.65, 4.7), II: 30.6 (8.85, 5.45, 5.75, 5.85, 4.7), III: 27.05 (7.3, 4.85, 4.5, 6.2, 4.2), IV: 36.85 (9.8, 6.25, 7.6, 8.4, 4.8). Metatarsal scopulae: I, II: 95%, III, IV: 75%. Distal tibial spines: I, III, IV: 1m; II: 1m1r. Distal ventral metatarsal spines: I, II: 1m1r; III, IV: 1p1m1r.

Spinnerets: PLS: basal article: 1.5 long, median article: 1.45 long, apical article: 2.5 long. PMS: 1.8 long.

Receptacles as in Fig. 21E; stalk short and narrow; apical lobes well-defined, outer lobe larger than inner lobe.

Colour in life (Fig. 23E): overall body and legs light golden brown.

###### Natural history.

**Figure 24.**
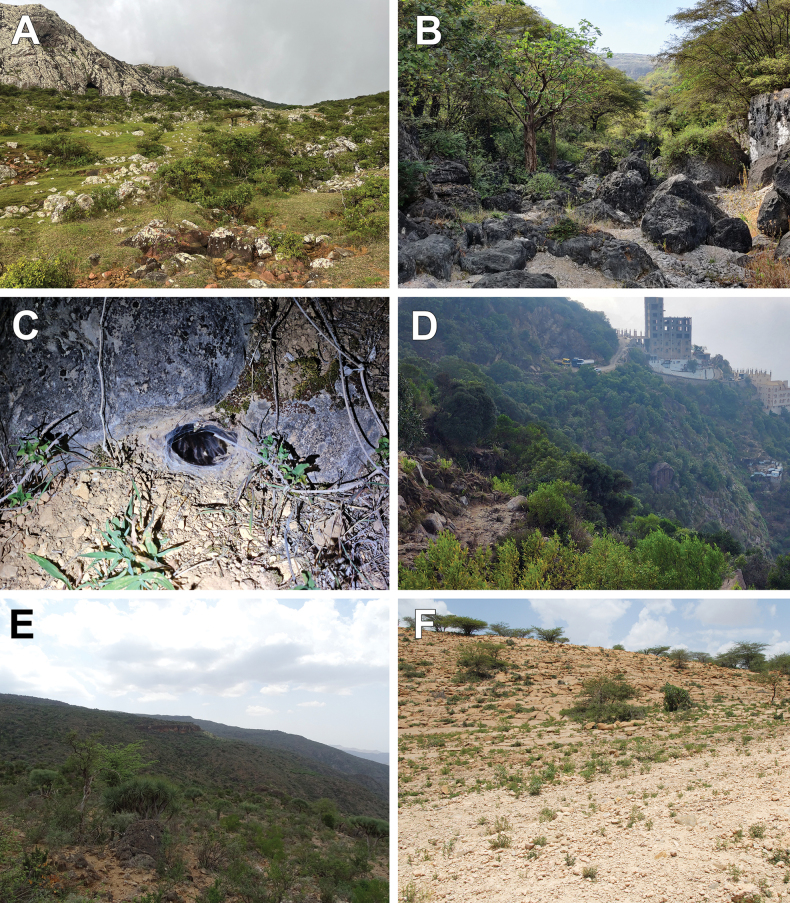
Habitats (**A, B, D–F**) and a female’s burrow (**C**). **A.***Monocentropusbalfouri* (Hajhir Mountains, Socotra, Yemen); **B, C.***Satyrexferox* sp. nov. (Sarfayt, Dhofar, Oman); **D.***S.arabicus* sp. nov. (Faifa Mountains, Jazan, Saudi Arabia); **E.***S.speciosus* sp. nov. (Daallo, Sanaag, Somaliland); **F.***S.somalicus* sp. nov. (Quljeed, Awdal, Somaliland). Photos by James Bailey (**A**), MS (**B, C**), Ibrahim Mohssin Fageeh (**D**), PJ (**E**), and František Kovařík (**F**).

A fossorial species that constructs deep burrows, lined only sparsely with silk, in clayey soil beneath deeply embedded limestone rocks. The habitat is a semi-arid montane shrubland with rocky slopes and sparse vegetation (Fig. 24E).

###### Distribution.

**Figure 25.**
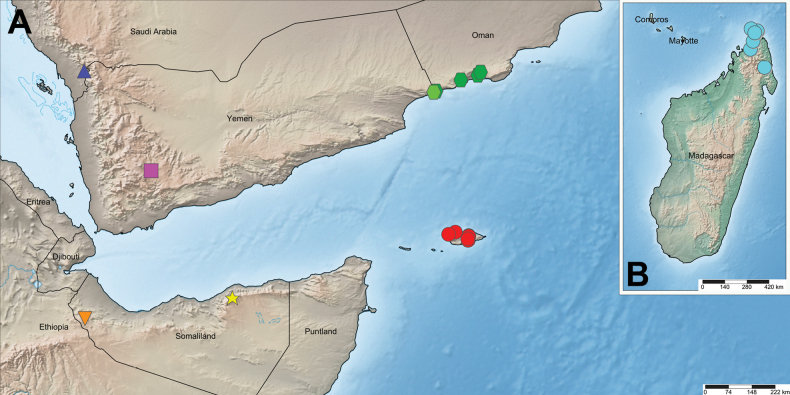
Distribution records of the species treated in this paper in the southern Arabian Peninsula and Somaliland (**A**), and Madagascar (**B**). Red circles: *Monocentropusbalfouri*, cyan circles: “*Monocentropus*” *lambertoni*, pale green: hexagon *Satyrexferox* sp. nov. (type locality), dark green: hexagons *S.ferox* sp. nov. (other localities), blue triangle: *S.arabicus* sp. nov., pink square: *S.longimanus* comb. nov., yellow star: *S.speciosus* sp. nov., orange inverted triangle: *S.somalicus* sp. nov.

Known only from the type locality in the Sanaag Region, northeastern Somaliland (Fig. 25).

##### ﻿ Satyrex somalicus

Taxon classificationAnimaliaAraneaeTheraphosidae

Zamani & von Wirth sp. nov.

3B2398E5-54FE-5C67-9442-0209DA88E58C

https://zoobank.org/8D2A34E5-224F-4EF5-9AA6-8F3790AD4515

Figs 4D, H , 5D, H , 8G , 10F , 13F , 14F , 15F , 16F , 17F , 18F , 19F , 20K, L

###### Type material.

***Holotype*** • ♂ (SMNS-Aran-004397), Somaliland: Awdal Reg.: Quljeed, 10°07'N, 43°00'E, 1300 m, 12.9.2017 (F. Kovařík).

###### Etymology.

The specific epithet refers to the distribution of the species in the Somali Peninsula (= the Horn of Africa).

###### Common name.

We propose “Somali dwarf tarantula” as a common name.

###### Diagnosis.

The male of *S.somalicus* sp. nov. resembles that of *S.speciosus* sp. nov. in the shape of the bulb. It differs in its smaller size (carapace length 9.35 vs 13.6), by the shorter palp (palp/carapace length ratio: 2.23 vs 3.13; cf. Fig. 13F vs Fig. 13E), the tegulum almost as long as wide (vs longer than wide), the retrolateral keel of the bulb well-developed (vs poorly developed), and the different curvature of the embolus (cf. Fig. 14F vs Fig. 14E).

###### Description.

**Male** (SMNS-Aran-004397). Habitus as in Figs 4D, H, 5D, H. Total length 18.3. Carapace 9.35 long, 8.35 wide. Eye tubercle as in Fig. 8G. Stridulatory organs of coxa and trochanter I as in Fig. 10F. Cheliceral furrow with ten promarginal teeth and six mesobasal denticles. Sternum 3.95 long, 3.5 wide. Labium and each maxilla with ~110 cuspules.

Measurements of palp and legs: palp: 20.9 (7.8, 4.15, 7.4, —, 1.55), I: 32.8 (8.95, 4.95, 7.6, 7.1, 4.2), II: 29.9 (8.55, 4.55, 6.1, 6.7, 4.0), III: 28.25 (8.0, 3.85, 4.45, 7.85, 4.1), IV: 30.7 (9.5, 6.6, 3.1, 7.3, 4.2). Full palp as in Fig. 13F; 2.23× longer than carapace. Tibial apophysis as in Fig. 20K, L; with 12 spines. Metatarsal scopulae: I, II: 95%, III: 55%, IV: 50%. Distal tibial spines: I: 2r; II: 1p1r; III, IV: 1r. Distal ventral metatarsal spines: I, II, III, IV: 1p1m1r.

Spinnerets: PLS: damaged, median and apical articles missing. PMS: 1.0 long.

Bulb as in Figs 14F, 15F, 16F, 17F; tegulum almost as long as wide; embolus sharply curved and tapering towards apex, tip with prolateral inferior (*PI*) and prolateral superior (*PS*) keels (Figs 18F, 19F).

Colour in life: greyish brown.

**Female.** Unknown.

###### Natural history.

The only collected specimen was found in a burrow excavated in compacted sandy-loam soil beneath a rock, in a semi-arid area with scattered acacia scrubland (Fig. 24F).

###### Comments.

The generic placement of this species is tentative and should be re-evaluated once additional material becomes available for detailed morphological examination and molecular analyses. Such analyses were not possible in the present study due to the poor preservation of the holotype, which was partially crushed shortly before collection. This species differs from others in the genus by its smaller size, much shorter palp, distinctly broadened apex of the paddle seta, and a very well-developed retrolateral keel.

###### Distribution.

Known only from the type locality in the Awdal Region, western Somaliland (Fig. 25).

## ﻿Discussion

Here, the genus *Monocentropus* was redefined as monotypic and endemic to Socotra Island. Although the generic placement of the Madagascar-endemic species “*Monocentropus*” *lambertoni* sensu lato, here considered a species complex, remains unclear, both morphological and molecular data confirm relatively close relationships between the Malagasy and Socotran lineages. Biogeographic connections between taxa from these two islands have been documented in several other groups, particularly reptiles. Gemsnakes of the family Pseudoxyrhophiidae Dowling, 1975 are one such example. As first noted by Nagy et al. (2003), the Socotran endemic *Ditypophisvivax* Günther, 1881 is closely related to a diverse radiation of colubroid snakes from Madagascar, whereas continental Africa is mainly populated by other colubroid clades. Subsequent studies (e.g., Burbrink et al. 2019) have shown that two African genera—*Amplorhinus* Smith, 1847 and *Duberria* Fitzinger, 1826—also belong to the Pseudoxyrhophiinae Dowling, 1975, and that the divergence between *Ditypophis* Günther, 1881 and the Malagasy radiation dates back to the Oligocene (Nagy et al. 2003; Burbrink et al. 2019). This supports the scenario proposed by Nagy et al. (2003), in which pseudoxyrhophiids once had a broader distribution across Africa but were largely replaced by other colubroids, surviving only on Socotra and Madagascar. It will be interesting to compare this pattern with the evolutionary history of eumenophorine tarantulas, for which molecular data remain scarce; divergence time estimates (Korba et al. 2022) suggest an Eocene–Oligocene diversification of the *Monocentropus* + *Satyrex* clade, similar to pseudoxyrhophiid snakes.

Other Socotran reptiles, however, show no biogeographic connection to Madagascar and instead originated from more recent dispersal events from Arabia or Africa. Based on current knowledge, skinks of the genus *Trachylepis* Fitzinger, 1843 colonised the island twice via overwater dispersal during the Miocene (Sindaco et al. 2012). Lizards of the genus *Mesalina* Gray, 1838 arrived on Socotra through long-distance dispersal from Arabia in the Miocene and subsequently diversified into two endemic species (Simó-Riudalbas et al. 2019). The Socotran chameleon, *Chamaeleomonachus* Gray, 1865, is sister to a clade of Arabian and North African species (Macey et al. 2008), with divergence estimated to have occurred in the early Miocene (Tolley et al. 2013). Geckos colonised the Socotra Archipelago independently on at least seven occasions and at different times (Garcia-Porta et al. 2016). For example, *Haemodracon* Bauer, Good & Branch, 1997 geckos diverged by vicariance from their African sister group in the Eocene and subsequently diversified on Socotra during the Miocene (Tamar et al. 2019), whereas *Hemidactylus* Oken, 1817 geckos dispersed from Arabia in two separate events during the Miocene and Pliocene (Šmíd et al. 2013). More comprehensive comparative studies of Socotran biota are needed to identify potential biogeographic connections between Socotra and Madagascar in other taxa, such as gentians (Yuan et al. 2005).

The other species originally described in *Monocentropus*, *M.longimanus*, was transferred to the newly established genus *Satyrex* gen. nov., which also includes four species newly described herein. All five species can be partially diagnosed by their elongated male palps, which appear to be the longest known among all tarantula species. In general, elongated male palps are uncommon in spiders, although genera exhibiting this trait, either in some or all of their species, are found in several families, such as Actinopodidae Simon, 1892 (all three genera), Ctenidae Keyserling, 1877 (*Sinoctenus* Marusik, Zhang & Omelko, 2012), Filistatidae Ausserer, 1867 (Filistatinae), Hypochilidae Marx, 1888 (*Ectatosticta* Simon, 1892), Mimetidae Simon, 1881 (e.g., *Gelanor* Thorell, 1869), Salticidae Blackwall, 1841 (e.g., *Viciria* Thorell, 1877), Tetragnathidae Menge, 1866 (e.g., *Leucauge* White, 1841), Theridiidae Sundevall, 1833 (e.g., *Rhomphaea* L. Koch, 1872), and Thomisidae Sundevall, 1833 (e.g., *Epidius* Thorell, 1877). The function of such elongation in palps remains unstudied in most groups. Amongst the groups noted above, the resting position of the male palp in Filistatinae resembles that in *Satyrex* gen. nov. the most, with the femur and tibia being held nearly parallel in both groups. Among Filistatinae, the species with the longest male palp relative to carapace length appears to be *Kukulcaniatractans* (O. Pickard-Cambridge, 1896), with a ratio of 4.3 (Magalhaes and Ramírez 2019). Filistatids are similar to mygalomorphs in lifestyle and general mating position (Barrantes and Ramírez 2013). During mating, male filistatids typically maintain a safe distance from the elevated female by pressing their first two pairs of legs against hers, thereby stabilising themselves before initiating copulation (Gerhardt 1928, 1944; Barrantes and Ramírez 2013).

Males of most theraphosids—excluding all genera within Selenocosmiinae Simon 1889 and a few representatives of other subfamilies, such as *Anoploscelus* Pocock, 1897 (Eumenophorinae), *Sericopelma* Ausserer, 1875 (Theraphosinae Thorell, 1869) and *Lampropelmacarpenteri* (Smith & Jacobi, 2015) (Ornithoctoninae Pocock 1895)—have a specialised apophysis on the distal ventral or proventral surface of tibia I (Pérez-Miles 2020). This structure is used to clasp the female’s chelicerae and elevate her, allowing the male to safely approach and copulate from a position beneath her. Males of Selenocosmiinae, which entirely lack a tibial apophysis, compensate with relatively elongated palps and adopt a mating position that involves sliding underneath the female during copulation, rather than lifting her to the extent seen in other subfamilies (VvW, pers. obs.).

In *Satyrex* gen. nov., males exhibit highly elongated palps despite the presence of a tibial apophysis. This combination appears to facilitate copulation while minimising the risk of cannibalism, as it allows the male to position himself slightly away from the female rather than directly beneath her. This is plausible given the extremely aggressive defence behaviour observed in females of this genus. These hypotheses, however, remain tentative and should be further evaluated through direct observation and documentation of mating behaviour in these tarantulas, which has not yet been possible.

## Supplementary Material

XML Treatment for Monocentropus

XML Treatment for Monocentropus balfouri

XML Treatment for "Monocentropus" lambertoni

XML Treatment for Satyrex

XML Treatment for Satyrex ferox

XML Treatment for Satyrex arabicus

XML Treatment for Satyrex longimanus

XML Treatment for Satyrex speciosus

XML Treatment for Satyrex somalicus

## ﻿Additional information

### Use of AI

No use of AI was reported.

### Author ORCIDs

Alireza Zamani https://orcid.org/0000-0002-8084-9666

Volker von Wirth https://orcid.org/0000-0003-2868-0768

Přemysl Fabiánek https://orcid.org/0009-0002-2147-7521

Jonas Höfling https://orcid.org/0009-0001-8664-8021

Pavel Just https://orcid.org/0000-0003-1983-6310

Jan Korba https://orcid.org/0009-0006-0578-1725

Alice Petzold https://orcid.org/0000-0002-0743-2004

Mark Stockmann https://orcid.org/0000-0003-2957-0181

Hassan Sh Abdirahman Elmi https://orcid.org/0000-0002-1810-8170

Miguel Vences https://orcid.org/0000-0003-0747-0817

Vera Opatova https://orcid.org/0000-0002-4324-1344

### Data availability

All of the data that support the findings of this study are available in the main text or Supplementary Information.

## Supplementary materials

Supplementary material 1 Bayesian consensus tree recovered by MrBayes with posterior probability values Data type jpg https://binary.pensoft.net/file/1379498This dataset is made available under the Open Database License (http://opendatacommons.org/licenses/odbl/1.0/). The Open Database License (ODbL) is a license agreement intended to allow users to freely share, modify, and use this Dataset while maintaining this same freedom for others, provided that the original source and author(s) are credited.Alireza Zamani, Volker von Wirth, Přemysl Fabiánek, Jonas Höfling, Pavel Just, Jan Korba, Alice Petzold, Mark Stockmann, Hassan Sh Abdirahman Elmi, Miguel Vences, Vera Opatova

Supplementary material 2 Maximum likelihood tree recovered by IQ-TREE with ultrafast bootstrap supports Data type jpg https://binary.pensoft.net/file/1379499This dataset is made available under the Open Database License (http://opendatacommons.org/licenses/odbl/1.0/). The Open Database License (ODbL) is a license agreement intended to allow users to freely share, modify, and use this Dataset while maintaining this same freedom for others, provided that the original source and author(s) are credited.Alireza Zamani, Volker von Wirth, Přemysl Fabiánek, Jonas Höfling, Pavel Just, Jan Korba, Alice Petzold, Mark Stockmann, Hassan Sh Abdirahman Elmi, Miguel Vences, Vera Opatova

Supplementary material 3 Detailed locality information and GenBank accession codes for samples used in this study Data type docx https://binary.pensoft.net/file/1379500This dataset is made available under the Open Database License (http://opendatacommons.org/licenses/odbl/1.0/). The Open Database License (ODbL) is a license agreement intended to allow users to freely share, modify, and use this Dataset while maintaining this same freedom for others, provided that the original source and author(s) are credited.Alireza Zamani, Volker von Wirth, Přemysl Fabiánek, Jonas Höfling, Pavel Just, Jan Korba, Alice Petzold, Mark Stockmann, Hassan Sh Abdirahman Elmi, Miguel Vences, Vera Opatova
