# Supplementary figures and images for: ﻿Size matters: a new genus of tarantula with the longest male palps, and an integrative revision of Monocentropus Pocock, 1897 (Araneae, Theraphosidae, Eumenophorinae)
Source: Zookeys. 2025 Jul 22;1247:89–126. doi: 10.3897/zookeys.1247.162886 (PMC12308207; doi:10.3897/zookeys.1247.162886)

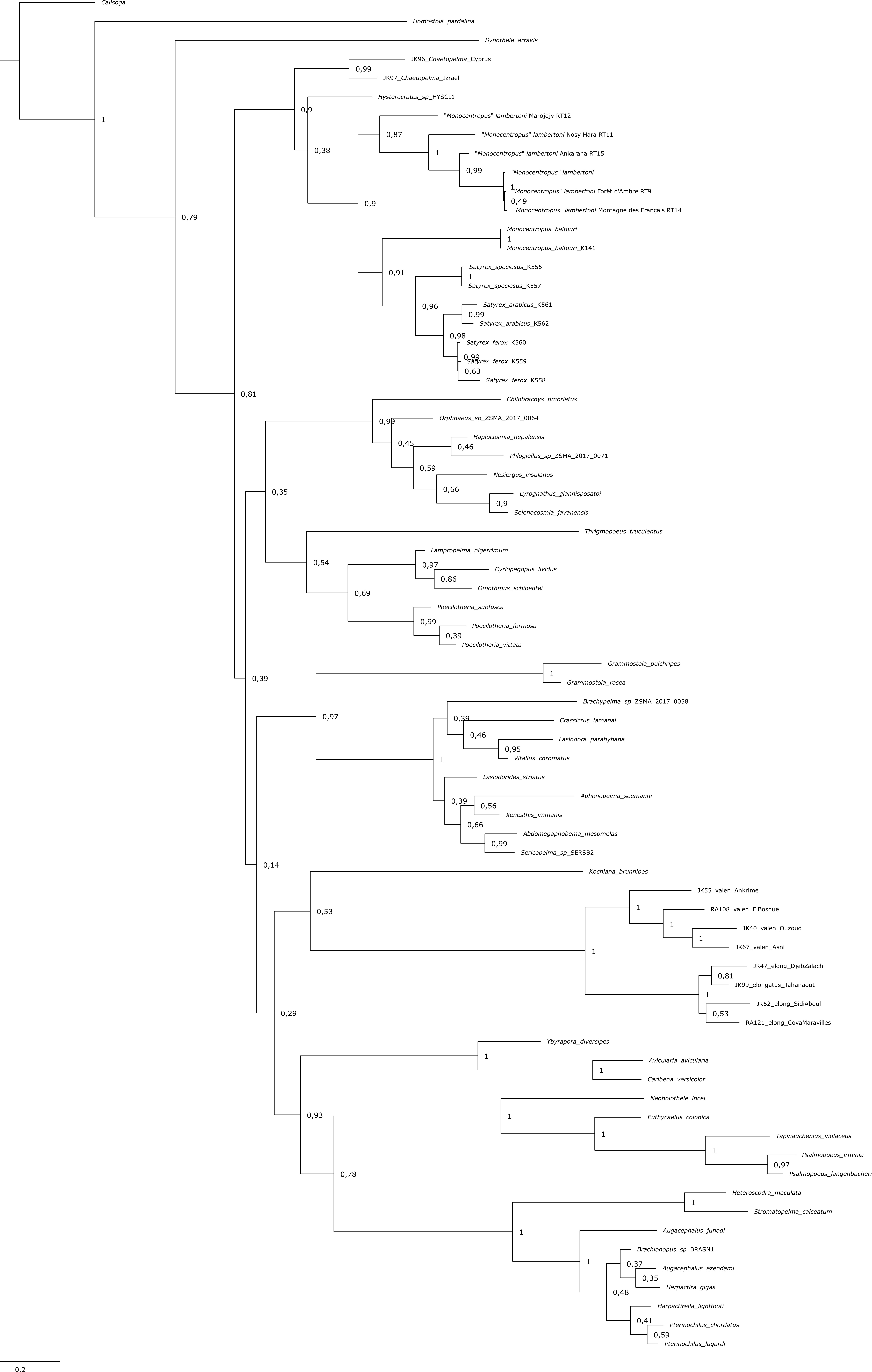

Supplement: Supplementary material 1 — Bayesian consensus tree recovered by MrBayes with posterior probability values [file zookeys-1247-089_article-162886__-s001.jpg]

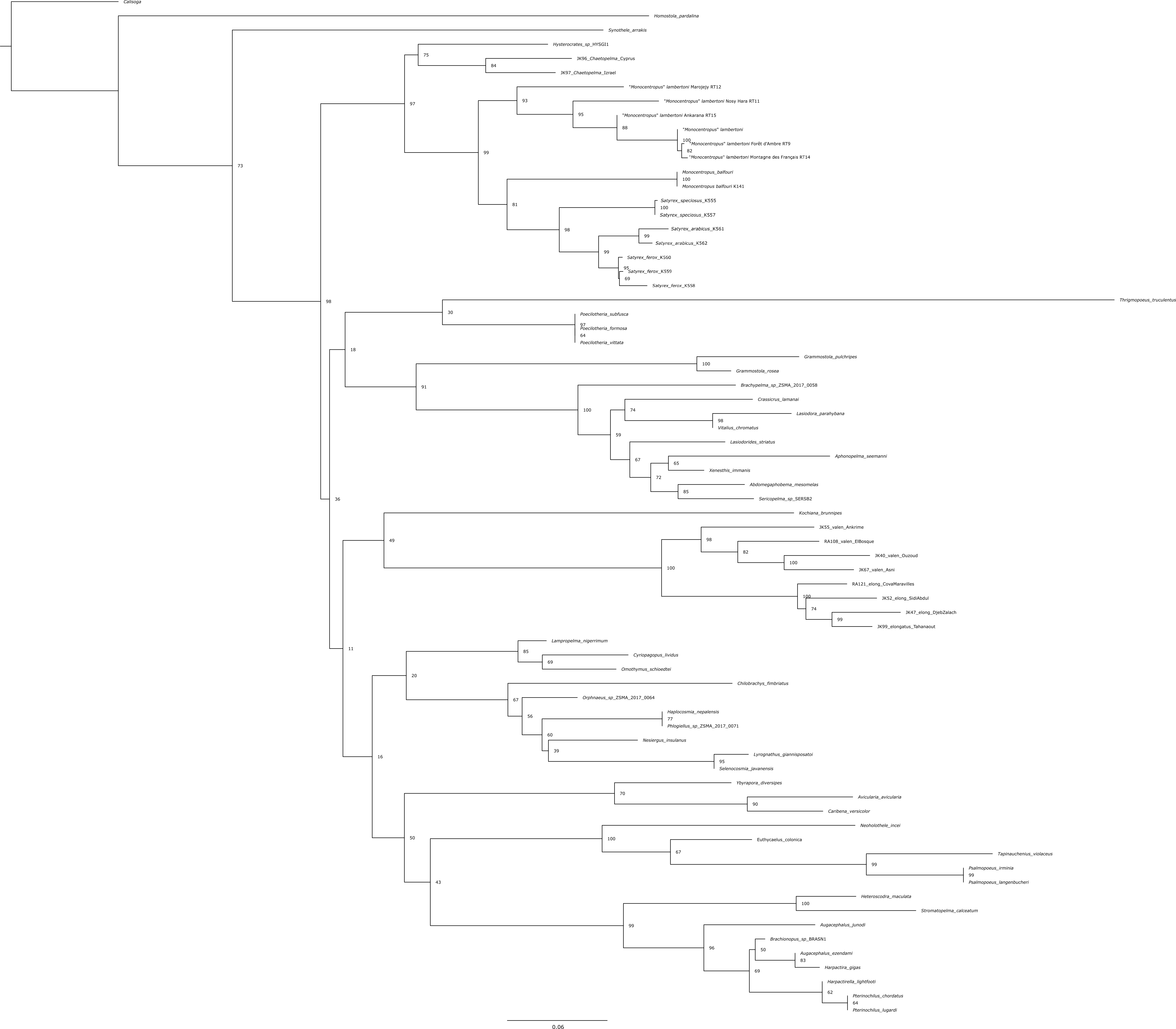

Supplement: Supplementary material 2 — Maximum likelihood tree recovered by IQ-TREE with ultrafast bootstrap supports [file zookeys-1247-089_article-162886__-s002.jpg]
