# Supplementary material for: ﻿Size matters: a new genus of tarantula with the longest male palps, and an integrative revision of Monocentropus Pocock, 1897 (Araneae, Theraphosidae, Eumenophorinae)
Source: Zookeys. 2025 Jul 22;1247:89–126. doi: 10.3897/zookeys.1247.162886 (PMC12308207; doi:10.3897/zookeys.1247.162886)
Supplement: Supplementary material 3 — Detailed locality information and GenBank accession codes for samples used in this study [file zookeys-1247-089_article-162886__-s003.docx]

**Table S1.** Detailed locality information and GenBank accession codes for samples used in this study.

| **DNA code** | **Collection code** | **Depository code** | **Genus** | **Species** | **Country** | **Locality** | **Lat.** | **Long.** | **COI** | **28S** | **18S** | **Reference** |
| --- | --- | --- | --- | --- | --- | --- | --- | --- | --- | --- | --- | --- |
| RT11 | FGZC 1333 * | - | “*Monocentropus*” | sp. | Madagascar | Nosy Hara | 12.2371°S | 49.0123°E | MG273519 | MG273635 | MG273591 | Lüddecke et al. (2018) |
| RT12 | ZCMV 2053 * | - | “*Monocentropus*” | sp. | Madagascar | Marojejy | 14.4376°S | 49.7755°E | MG273532 | MG273644 | MG273609 | Lüddecke et al. (2018) |
| RT14 | - | - | “*Monocentropus*” | sp. | Madagascar | Montagne des Français | 12.3233°S | 49.3386°E | PV939177 | PV940022 | PV940025 | this study |
| RT9 | FGZC 1854 * | - | “*Monocentropus*” | sp. | Madagascar | Forêt d’Ambre | 12.4584°S | 49.2244°E | PV939176 | PV940021 | PV940024 | this study |
| RT15 | - | - | “*Monocentropus*” | sp. | Madagascar | Ankarana | 12.9336°S | 49.1268°E | - | PV940023 | PV940026 | this study |
| MygalGBOL098 | - | - | “*Monocentropus*” | *lambertoni* | Madagascar | Unknown | - | - | MG273529 | - | MG273603 | Lüddecke et al. (2018) |
| - | - | *-* | *Monocentropus* | *balfouri* | Yemen | Socotra (pet trade) | - | - | MG273530 | - | MG273605 | Lüddecke et al. (2018) |
| K141 | MGK00320 | - | *Monocentropus* | *balfouri* | Yemen | Socotra | - | - | PV942504 | PV940937 | PV940931 | this study |
| K558 | - | SMNS-Aran-004391 | *Satyrex* | *ferox* | Yemen | NE Al Ghaydah | 16°39'N | 53°03'E | - | PV940940 | - | this study |
| K559 | - | SMNS-Aran-004389 | *Satyrex* | *ferox* | Yemen | NE Al Ghaydah | 16°39'N | 53°03'E | PV942507 | PV940941 | PV940934 | this study |
| K560 | - | SMNS-Aran-004392 | *Satyrex* | *ferox* | Yemen | NE Al Ghaydah | 16°39'N | 53°03'E | PV942508 | PV940942 | PV940935 | this study |
| K555 | - | SMNS-Aran-004396 | *Satyrex* | *speciosus* | Somaliland | Daallo | 10°48'N | 47°19'E | PV942505 | PV940938 | PV940932 | this study |
| K557 | - | SMNS-Aran-004395 | *Satyrex* | *speciosus* | Somaliland | Daallo | 10°48'N | 47°19'E | PV942506 | PV940939 | PV940933 | this study |
| K561 | - | SMNS-Aran-004393 | *Satyrex* | *arabicus* | Saudi Arabia | Jazan | 17°15'N | 43°06'E | PV942509 | PV940943 | PV940936 | this study |
| K562 | - | SMNS-Aran-004394 | *Satyrex* | *arabicus* | Saudi Arabia | Jazan | 17°15'N | 43°06'E | PV942510 | PV940944 | - | this study |
| JK96 | CRBAMM000313 | - | *Chaetopelma* | sp. | Cyprus | Protaras | - | - | OK428792 | OM618700 | OM618706 | Korba et al. (2022) |
| JK97 | CRBAMM000315 | - | *Chaetopelma* | sp. | Israel | Mont Hermon | - | - | OK428793 | OM618701 | OM618707 | Korba et al. (2022) |
| - | - | - | *Tliltocatl* | *vagans* | - | - | - | - | AJ584636 | - | - | Longhorn et al. (2007) |
| - | - | - | *Calisoga* | sp. | - | - | - | - | KR182728 | DQ639830 | DQ639925 | Hedin and Bond (2007)  Leavitt et al. (2015) |
| - | - | - | *Homosotola* | *pardalina* | - | - | - | - | - | KY017017 | - | Wheeler et al. (2016) |
| - | - | - | *Synothele* | *arrakis* | - | - | - | - | KY017602 | KY016947 | DQ639777 | Hedin and Bond (2007)  Wheeler et al. (2016) |
| - | - | - | *Phlogielus* | sp. | - | - | - | - | MG273510 | MG273626 | MG273581 | Lüddecke et al. (2018) |
| - | - | - | *Ybyrapora* | *diversipes* | - | - | - | - | MG273533 | - | - | Lüddecke et al. (2018) |
| - | - | - | *Thrigmopoeus* | *truculentus* | - | - | - | - | - | - | MG273604 | Lüddecke et al. (2018) |
| - | - | - | *Tapinauchenius* | *violaceus* | - | - | - | - | - | MG273615 | MG273568 | Lüddecke et al. (2018) |
| - | - | - | *Nesiergus* | *insulanus* | - | - | - | - | MG273531 | MG273643 | - | Lüddecke et al. (2018) |
| - | - | - | *Lasiodorides* | *striatus* | - | - | - | - | MG273509 | MG273623 | MG273578 | Lüddecke et al. (2018) |
| - | - | - | *Lampropelma* | *nigerrimum* | - | - | - | - | MG273506 | MG273620 | MG273574 | Lüddecke et al. (2018) |
| - | - | - | *Haplocosmia* | *nepalensis* | - | - | - | - | - | - | MG273608 | Lüddecke et al. (2018) |
| - | - | - | *Euthycaleus* | *colonica* | - | - | - | - | MG273507 | - | MG273576 | Lüddecke et al. (2018) |
| - | - | - | *Caribena* | *versicolor* | - | - | - | - | MG273534 | MG273646 | MG273611 | Lüddecke et al. (2018) |
| - | - | - | *Linothele* | sp. | - | - | - | - | - | MG273624 | MG273579 | Lüddecke et al. (2018) |
| - | - | - | *Sericopelma* | sp. | - | - | - | - | MG273514 | MG273630 | MG273586 | Lüddecke et al. (2018) |
| - | - | - | *Hysterocrates* | sp. | - | - | - | - | MG273528 | - | MG273602 | Lüddecke et al. (2018) |
| - | - | - | *Brachypelma* | sp. | - | - | - | - | MG273502 | MG273617 | MG273570 | Lüddecke et al. (2018) |
| - | - | - | *Selenocosmia* | *javanensis* | - | - | - | - | MG273512 | MG273628 | MG273584 | Lüddecke et al. (2018) |
| - | - | - | *Psalmopoeus* | *langenbucheri* | - | - | - | - | - | - | MG273607 | Lüddecke et al. (2018) |
| - | - | - | *Psalmopoeus* | *irminia* | - | - | - | - | MG273511 | MG273627 | MG273582 | Lüddecke et al. (2018) |
| - | - | - | *Poecilotheria* | *vittata* | - | - | - | - | - | - | MG273567 | Lüddecke et al. (2018) |
| - | - | - | *Poecilotheria* | *subfusca* | - | - | - | - | - | - | MG273606 | Lüddecke et al. (2018) |
| - | - | - | *Vitalius* | *chromatus* | - | - | - | - | MG273501 | MG273614 | MG273566 | Lüddecke et al. (2018) |
| - | - | - | *Abdomegaphobema* | *mesomelas* | - | - | - | - | MG273513 | MG273629 | MG273585 | Lüddecke et al. (2018) |
| - | - | - | *Kochiana* | *brunnipes* | - | - | - | - | MG273508 | MG273622 | MG273577 | Lüddecke et al. (2018) |
| - | - | - | *Harpactirella* | *lightfooti* | - | - | - | - | MG273521 | MG273636 | MG273593 | Lüddecke et al. (2018) |
| - | - | - | *Harpactira* | *gigas* | - | - | - | - | MG273524 | - | MG273598 | Lüddecke et al. (2018) |
| - | - | - | *Grammostola* | *pulchripes* | - | - | - | - | MG273517 | MG273633 | MG273589 | Lüddecke et al. (2018) |
| - | - | - | *Cyriopagopus* | *lividus* | - | - | - | - | MG273504 | MG273619 | MG273572 | Lüddecke et al. (2018) |
| - | - | - | *Chilobrachys* | *fimbriatus* | - | - | - | - | MG273503 | MG273618 | MG273571 | Lüddecke et al. (2018) |
| - | - | - | *Augacephalus* | *ezendami* | - | - | - | - | MG273522 | MG273637 | MG273594 | Lüddecke et al. (2018) |
| - | - | - | *Xenesthis* | *immanis* | - | - | - | - | MG273518 | MG273634 | MG273590 | Lüddecke et al. (2018) |
| - | - | - | *Neoholothele* | *incei* | - | - | - | - | MG273505 | - | MG273573 | Lüddecke et al. (2018) |
| - | - | - | *Crassicrus* | *lamanai* | - | - | - | - | MG273520 | - | MG273592 | Lüddecke et al. (2018) |
| - | - | - | *Poecilotheria* | *formosa* | - | - | - | - | MG273516 | MG273632 | MG273588 | Lüddecke et al. (2018) |
| - | - | - | *Augacephalus* | *junodi* | - | - | - | - | MG273523 | MG273640 | MG273597 | Lüddecke et al. (2018) |
| - | - | - | *Aphonopelma* | *seemani* | - | - | - | - | - | MG273616 | MG273569 | Lüddecke et al. (2018) |
| - | - | - | *Omothymus* | *schioedtei* | - | - | - | - | MG273500 | MG273613 | MG273565 | Lüddecke et al. (2018) |
| - | - | - | *Avicularia* | *avicularia* | - | - | - | - | MG273535 | MG273647 | MG273612 | Lüddecke et al. (2018) |
| - | - | - | *Grammostola* | *rosea* | - | - | - | - | MG273515 | MG273631 | MG273587 | Lüddecke et al. (2018) |
| - | - | - | *Stromatopelma* | *calceatum* | - | - | - | - | MG273526 | - | - | Lüddecke et al. (2018) |
| - | - | - | *Heteroscodra* | *maculata* | - | - | - | - | MG273525 | - | MG273599 | Lüddecke et al. (2018) |

* ZCMV and FGZC are field numbers assigned by Frank Glaw and Miguel Vences, respectively; the specimens will be incorporated into the collection of the Zoologische Staatssammlung München in Munich, Germany.

**References:**

Hedin M, Bond JE (2006) Molecular phylogenetics of the spider infraorder Mygalomorphae using nuclear rRNA genes (18S and 28S): Conflict and agreement with the current system of classification. Molecular Phylogenetics and Evolution 41(2): 454–471. <https://doi.org/10.1016/j.ympev.2006.05.017>

Leavitt DH, Starrett J, Westphal MF, Hedin M (2015) Multilocus sequence data reveal dozens of putative cryptic species in a radiation of endemic Californian mygalomorph spiders (Araneae, Mygalomorphae, Nemesiidae). Molecular Phylogenetics and Evolution 91: 56–67. <https://doi.org/10.1016/j.ympev.2015.05.016>

Longhorn SJ, Nicholas M, Chuter J, Vogler AP (2007) The utility of molecular markers from non-lethal DNA samples of the CITES II protected “tarantula” *Brachypelma vagans* (Araneae, Theraphosidae). The Journal of Arachnology. 35: 278–292. <https://doi.org/10.1636/S05-62.1>

Lüddecke T, Krehenwinkel H, Canning G, Glaw F, Longhorn SJ, Taenzler R, Wendt I, Vences M (2018) Discovering the silk road: Nuclear and mitochondrial sequence data resolve the phylogenetic relationships among theraphosid spider subfamilies. Molecular Phylogenetics and Evolution. 119: 63–70. <https://doi.org/10.1016/j.ympev.2017.10.015>

Wheeler WC, Coddington JA, Crowley LM, Dimitrov D, Goloboff PA, Griswold CE, Hormiga G, Prendini L, Ramirez MJ, Sierwald P, Almeida-Silva L, Alvarez-Padilla F, Arnedo MA, Benavides Silva LR, Benjamin SP, Bond JE, Grismado CJ, Hasan E, Hedin M, Izquierdo MA, Labarque FM, Ledford J, Lopardo L, Maddison WP, Miller JA, Piacentini LN, Platnick NI, Polotow D, Silva-Davila D, Scharff N, Szuts T, Ubick D, Vink CJ, Wood HM, Zhang J (2017) The spider tree of life: phylogeny of Araneae based on target-gene analyses from an extensive taxon sampling. Cladistics. 33: 574–61. <https://doi.org/10.1016/j.ympev.2017.10.015>
